# Cardiac remodeling after bariatric surgery associates with systemic metabolic reprogramming: a longitudinal untargeted metabolomics study

**DOI:** 10.1186/s12933-026-03231-y

**Published:** 2026-06-02

**Authors:** Carmine Izzo, Martina Lombardi, Maria Rosaria Rusciano, Alessio Trotta, Cristina Gatto, Valeria Visco, Albino Carrizzo, Mario Masarone, Marcello Persico, Vincenzo Pilone, Luigi Schiavo, Carmine Vecchione, Jacopo Troisi, Michele Ciccarelli

**Affiliations:** 1 Department of Medicine, Surgery and Dentistry, “Scuola Medica Salernitana”, University of Salerno, 84081 Baronissi, SA Italy; 2 Theoreo Srl Via Degli Ulivi, 3, 84090 Montecorvino Pugliano, SA Italy; 3 European Institute of Metabolomics (EIM) Foundation, Via G. Puccini, 2, 84081 Baronissi, SA Italy; 4 IRCCS Neuromed Mediterranean Neurological Institute, 86077 Pozzilli, Italy; 5 Public Health Department, Naples “Federico II” University, AOU “Federico II”, Via S. Pansini 5, 80131 Naples, Italy

**Keywords:** Untargeted metabolomics, Cardiac remodeling, Bariatric surgery, Weight loss, PLS-DA multivariate analysis, Metabolic signatures, Obesity-associated cardiomyopathy, Metabolic stratification, Precision cardiology

## Abstract

**Background:**

Obesity is a major contributor to adverse cardiac remodeling, particularly concentric left ventricular remodeling (LVCR), which is associated with diastolic dysfunction and increased cardiovascular risk. Bariatric surgery reverses many obesity-related comorbidities, including structural myocardial alterations. However, the molecular underpinnings of cardiac recovery, particularly metabolomic correlates, remain poorly understood.

**Objective:**

This study aimed to characterize longitudinal changes in the serum metabolome of patients undergoing bariatric surgery and to investigate the relationship between these metabolic shifts and the regression of concentric cardiac remodeling.

**Methods:**

A cohort of 127 adults with severe obesity was evaluated at baseline and 4, 12, and 24 weeks post-surgery. Echocardiographic parameters were assessed to define cardiac remodeling. Untargeted GC–MS-based metabolomic profiling was performed on serum samples. Multivariate models (PLS-DA), LASSO regression, and pathway enrichment analyses were employed to identify discriminant metabolites and predictors of remodeling outcomes.

**Results:**

Changes in cardiac geometry primarily occurred within the first 12 weeks after the intervention and were mainly driven by reductions in relative wall thickness (RWT). Left ventricular mass index (LVMI) showed only modest, non-significant changes at the cohort level, consistent with the predominantly non-hypertrophic baseline profile. Metabolomic profiling showed time-dependent shifts involving amino acid, fatty acid, and ketone metabolism. Patients who experienced early favorable cardiac remodeling after bariatric surgery demonstrated enriched signatures of mitochondrial substrate flexibility, including short-chain fatty acids, branched-chain amino acid metabolites, and dicarboxylic acids. LASSO regression identified specific sets of metabolites associated with reductions in LVMI and RWT.

**Conclusion:**

Early geometric cardiac adaptation following bariatric surgery—primarily reflected by reductions in relative wall thickness (RWT), with modest and non-significant changes in LVMI at the cohort level—is associated with coordinated changes in systemic metabolic profiles. These findings suggest potential metabolic correlates of early cardiac adaptation while highlighting substantial interindividual variability. Metabolomic profiling provides insights into individual metabolic trajectories and may inform future strategies for cardiometabolic risk stratification.

**Supplementary Information:**

The online version contains supplementary material available at 10.1186/s12933-026-03231-y.

## Research insights

**What is currently known about this topic?**

- Bariatric surgery reverses obesity-related cardiac remodeling
- Cardiac improvement often precedes maximal weight loss
- Metabolomics captures systemic changes after bariatric surgery

**What is the key research question?**

- Do metabolomic profiles explain heterogeneous cardiac remodeling after bariatric surgery?

**What is new?**

- Early cardiac remodeling associates with distinct metabolic trajectories
- Baseline metabolomic fingerprints could predict remodeling response
- Remodeling links to mitochondrial and substrate flexibility pathways

**How might this study influence clinical practice?**

- Metabolomics may contribute to future strategies for cardiometabolic risk stratification, pending validation in larger studies

## Introduction 

Obesity has emerged as a global pandemic and a major modifiable risk factor for a spectrum of non-communicable illnesses, particularly cardiovascular diseases (CVD), which remain the leading cause of morbidity and mortality worldwide. Defined by excessive accumulation of adipose tissue, obesity contributes to a complex pathophysiological environment characterized by chronic low-grade inflammation, insulin resistance, dyslipidemia, endothelial dysfunction, and hypertension [1], all of which converge to exacerbate cardiovascular risk and promote adverse cardiac remodeling [2].

A particularly insidious manifestation of obesity-induced cardiac damage is concentric left ventricular remodeling (LVR), a subtype of cardiac remodeling characterized by increased wall thickness and relative wall mass with preserved or even reduced chamber volume. This concentric remodeling is strongly associated with diastolic dysfunction and a heightened risk of heart failure with preserved ejection fraction (HFpEF) [3], as well as arrhythmogenesis due to changes in cardiac electrophysiological properties and autonomic modulation [4]. Emerging data underscore that such structural alterations are already present in obese individuals even in the absence of overt cardiovascular disease, qualifying them as early markers of subclinical cardiac dysfunction [5].

Bariatric surgery remains the most effective long-term treatment for severe obesity and its metabolic complications. Procedures such as Roux-en-Y gastric bypass (RYGB), sleeve gastrectomy (SG), and one-anastomosis gastric bypass (OAGB) are not only associated with substantial and sustained weight loss but also significant improvements in glucose metabolism, blood pressure, lipid profile, and systemic inflammatory markers [6]. Importantly, accumulating evidence indicates that bariatric surgery also produces meaningful structural and functional improvements in the myocardium, including reductions in left ventricular mass, reversal of concentric remodeling, and enhancement of both systolic and diastolic function [7, 8].

The regression of cardiac concentric remodeling following surgical weight loss has been consistently demonstrated across different populations and time scales. In a prospective cardiac magnetic resonance study, Jhaveri et al. documented a 32% reduction in left ventricular mass within 17 months post-bariatric surgery, which was linearly associated with reductions in BMI [9]. Similar echocardiographic data demonstrate normalization of previously hypertrophied myocardium and recovery of contractile reserve in severely obese patients after intervention [10].

Furthermore, obesity-related concentric remodeling is increasingly recognized not only as a consequence of hemodynamic overload but also as an indicator of impaired myocardial metabolic flexibility [11].

Normally, the adult heart demonstrates remarkable substrate adaptability, switching between fatty acids, glucose, ketone bodies, and amino acids to generate ATP efficiently based on energy demands [12].

However, in obesity and insulin-resistant states, this flexibility declines over time. The myocardium becomes metabolically inflexible, characterized by excessive reliance on fatty acid oxidation, mitochondrial dysfunction, accumulation of toxic lipid intermediates, increased oxidative stress, and decreased tricarboxylic acid (TCA) cycle activity. These changes promote hypertrophic signaling, remodeling of the extracellular matrix, and a higher relative wall thickness (RWT), leading to concentric geometric patterns and diastolic dysfunction [13, 14].

Bariatric surgery presents a unique human model of sudden systemic metabolic unloading. Beyond just weight loss, it triggers rapid changes in endocrine, inflammatory, and substrate processes that may help restore metabolic flexibility at both the systemic and myocardial levels. Therefore, the regression of concentric remodeling after bariatric surgery isn't only proportional to weight loss but also involves coordinated reprogramming of systemic metabolic pathways related to mitochondrial substrate use, amino acid turnover, short-chain fatty acid metabolism, and TCA cycle activity.

In this context, beyond anthropometric and echocardiographic measures, an emerging area in understanding the systemic effects of obesity and its surgical treatment is metabolomics—the large-scale profiling of small molecules and metabolic intermediates in biological fluids [15]. Metabolomic analyses provide a dynamic view of metabolic homeostasis and pathophysiology, allowing for the identification of biomarkers and pathways involved in cardiovascular disease, insulin resistance, hepatic steatosis, and inflammation [16]. Bariatric surgery has been shown to cause significant changes in metabolomic profiles, including decreases in branched-chain amino acids (BCAAs), trimethylamine-N-oxide (TMAO), phenylalanine, and other cardiotoxic metabolites linked to negative cardiovascular outcomes [16]. These changes not only relate to weight loss but also independently predict better insulin sensitivity and remission of type 2 diabetes mellitus [17]. Additionally, recent research has emphasized the potential of metabolomics to distinguish responders from non-responders regarding cardiovascular remodeling, highlighting its role as a precision medicine tool [18].

Therefore, this study was designed to combine longitudinal echocardiographic phenotyping with untargeted serum metabolomics to investigate whether different metabolic patterns contribute to diverse cardiac remodeling responses after bariatric surgery. By framing cardiac geometric adaptation within the concept of metabolic flexibility and mitochondrial efficiency, we aimed to examine the mechanistic link between systemic metabolomic changes and myocardial structural recovery.

## Methods

### Study design and population

This study is a prospective observational cohort analysis of obese patients who were candidates for sleeve gastrectomy (SG) at the University Hospital San Giovanni di Dio e Ruggi d’Aragona,Salerno. Patient recruitment occurred between June 2022 and April 2023, following the criteria established by the Italian Society of Surgery (SIC) and the Italian Society for Obesity Surgery (SICOB, [19]). All referred patients meeting these criteria were included, regardless of age. Patients with a history of major cardiovascular events or other significant current or past illnesses in the last 6 months before enrollment, such as cancer, were excluded.

Data collection occurred during the preoperative evaluation, which was conducted on a day-hospital basis at the same institution. Information gathered included anthropometric data, baseline vital signs, instrumental measurements, electrocardiograms, and transthoracic echocardiography. Laboratory parameters were obtained through the hospital’s electronic medical records system.

**Fig. 1:**
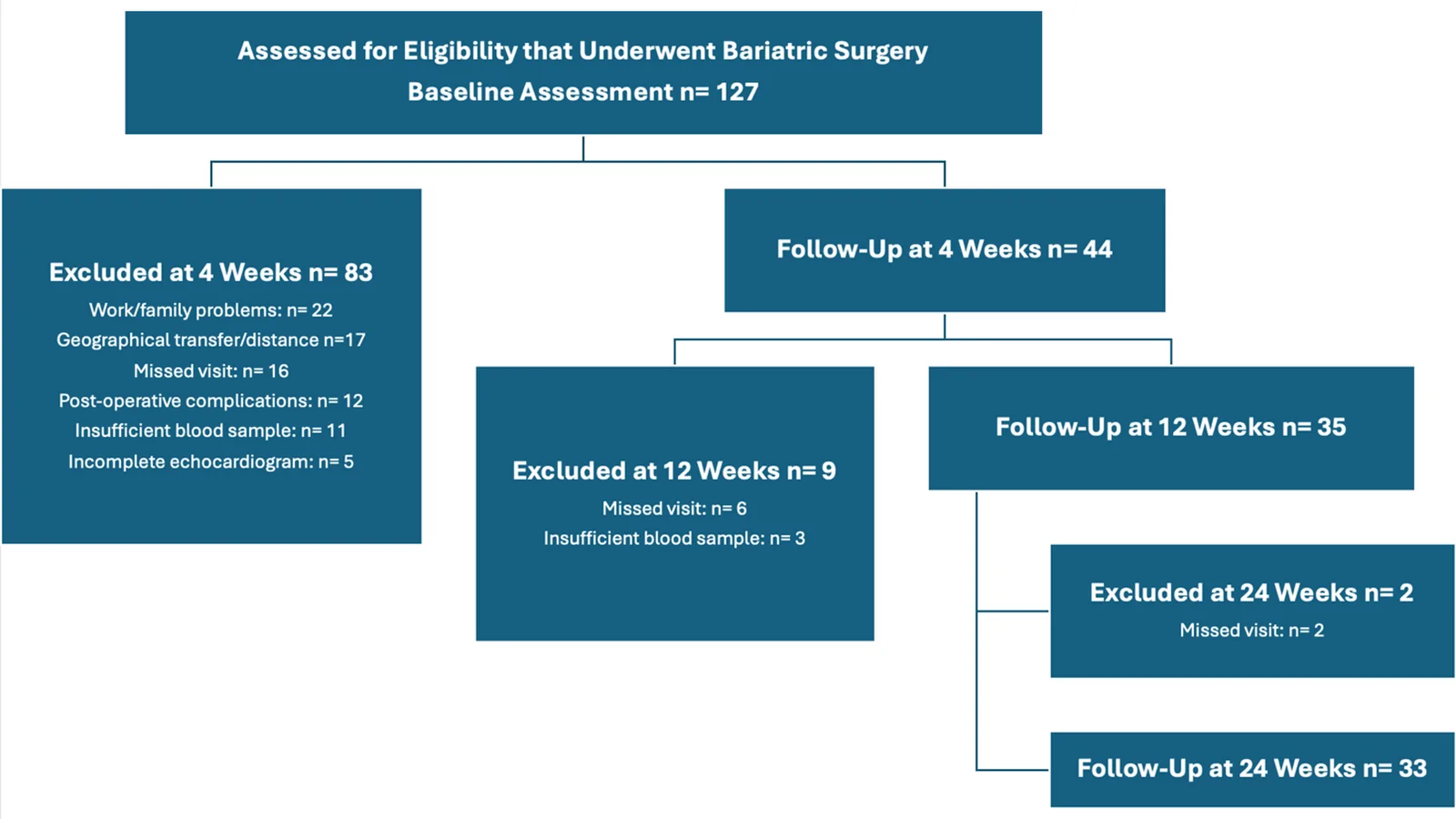
Participant flow diagram and CONSORT-style summary of cohort availability across study time points. Flow diagram illustrating participant progression through the longitudinal study evaluating cardiac remodeling and systemic metabolomic changes following bariatric surgery. A total of 127 patients were enrolled and underwent baseline clinical, echocardiographic, and metabolomic assessment. The diagram details the number of patients with available echocardiographic and plasma metabolomic samples at each follow-up time point (4, 12, and 24 weeks), together with reasons for missing data

A detailed overview of participant availability across time points, including completeness of metabolomic, echocardiographic, and laboratory data, as well as reasons for missingness, is provided in the participant flow diagram (Fig. 1). A schematic overview of the study design and analytical workflow is provided in Supplementary Fig. S0.

### Clinical examination and laboratory testing

For all enrolled patients, the following parameters were recorded: age, sex, height, weight, waist and hip circumferences, and body mass index (BMI). Height was measured with a wall-mounted stadiometer while patients stood barefoot with feet together. Weight was obtained using a KERN platform scale (model MPO 300 k-1LM, Frankfurt am Main, Germany), with patients dressed in light clothing and without footwear. Waist circumference was assessed using a rigid measuring tape positioned midway between the lower rib margin and the iliac crest, encircling the abdomen. Hip circumference was measured with a standard non-stretchable tape placed around the widest portion of the hips. BMI was calculated by dividing body weight in kilograms by the square of height in meters (kg/m^2^).

Blood pressure readings were taken three times over a 10–20-min period using the same aneroid sphygmomanometer (ERKA, model 1-tube EN 1060 Kobold Smart Rapid, Adult Large cuff 34–43 cm, Bad Tölz, Germany). Measurements were obtained with the cuff covering two-thirds of the left upper arm while patients were seated. The average of the three readings was used for analysis. Systolic blood pressure was determined at the onset of Korotkoff sounds, and diastolic pressure at the fifth Korotkoff phase. Results were expressed in mmHg.

Electrocardiography (ECG) was performed using standard 12-lead techniques with a MAC2000 device (GE Healthcare, Waukesha, WI, USA) to evaluate cardiac electrical function, and experienced clinicians interpreted tracings.

Fasting blood samples were analyzed to determine levels of triglycerides (TGs), total cholesterol, low-density (LDL) and high-density lipoproteins (HDL), fasting glucose, glycated hemoglobin (HbA1c), complete blood count (CBC), hemoglobin, C-reactive protein (CRP), erythrocyte sedimentation rate (ESR), N-terminal pro-B-type natriuretic peptide (NT-proBNP), albumin, creatinine, urea, uric acid, sodium, potassium, calcium, aspartate aminotransferase (AST), alanine aminotransferase (ALT), and total bilirubin. All laboratory biochemical analyses were performed at the clinical laboratory of Gaetano Fucito Hospital in Mercato San Severino (SA, Italy).

### Echocardiography and definitions of left ventricle remodeling and geometry

Transthoracic echocardiography was employed to assess ventricular morphology and performance using standard protocols. Two experienced cardiologists, both serving as medical directors and not blinded to patient status, conducted the examinations using a Vivid E9 ultrasound system (GE Healthcare, Waukesha, WI, USA). Offline analyses were performed using EchoPac software (version 201, GE Healthcare, Waukesha, WI, USA) with a 4.6 MHz transducer (GE-M5Sc-D XDClear, GE Medical Systems, Waukesha, WI, USA), ensuring consistency between assessments. Echocardiography allowed for precise evaluation of left ventricular (LV) remodeling. Measurements included interventricular septal thickness (IVS), posterior wall thickness (PW), and LV end-diastolic diameter (LVEDd), which were used to compute ventricular volume and mass according to standardized formulas. In accordance with the American Society of Echocardiography (ASE) recommendations, left ventricular mass (LVM) was indexed to body surface area (BSA), with normal thresholds set at < 95 g/m^2^ for women and < 115 g/m^2^ for men. LV hypertrophy (LVH) was defined by indexed values exceeding these cutoffs. RWT was calculated as (2× posterior wall thickness) / LV end-diastolic diameter and used to categorize the type of hypertrophy: concentric (RWT > 0.42) or eccentric (RWT ≤ 0.42). Additionally, concentric remodeling was identified in patients with a normal LVM index but elevated RWT.

Based on these parameters, four geometric LV patterns were recognized:

1. Normal geometry: LVMI ≤ 115 g/m^2^ (men), ≤ 95 g/m^2^ (women) and RWT ≤ 0.42
2. Concentric remodeling: LVMI ≤ 115 g/m^2^ (men), ≤ 95 g/m^2^ (women) and RWT > 0.42
3. Eccentric hypertrophy: LVMI > 115 g/m^2^ (men), > 95 g/m^2^ (women) and RWT ≤ 0.42
4. Concentric hypertrophy: LVMI > 115 g/m^2^ (men), > 95 g/m^2^ (women) and RWT > 0.42 [20]

Remodeling phenotypes were defined based on early changes in RWT at 4 weeks (T4) to capture initial geometric adaptation following bariatric surgery. This early classification was intended as an exploratory approach to investigate metabolic signatures associated with early structural changes and should not be interpreted as a definitive assessment of long-term cardiac remodeling. Accordingly, throughout the manuscript, the term “cardiac remodeling” refers to geometry-based classification derived from combined LVMI–RWT criteria, with early RWT-based subgrouping considered exploratory and hypothesis-generating.

For selected exploratory analyses, a binary LVMI-based classification (normal vs increased LVMI) was also applied to isolate the contribution of myocardial mass independent of geometric pattern. This secondary categorization was used specifically in analyses aimed at evaluating mass-dependent metabolomic associations and should not be interpreted as a replacement for the full geometric classification framework.

Unless otherwise specified, the term “cardiac remodeling” in this manuscript refers to deviations from normal geometry as defined by the combined LVMI–RWT classification.

### Untargeted metabolomics analysis

#### Sample preparation and extraction protocol

Untargeted profiling of serum metabolites was performed using the MetaboPrep GC extraction kit (Theoreo, Montecorvino Pugliano, Italy), following the manufacturer's specified protocol. A volume of 50 µL from each serum specimen was combined with the extraction buffer and an internal standard in microcentrifuge tubes. The resulting mixture underwent vortex agitation for 30 s at 1250 rpm and was subsequently centrifuged at 16,000 rpm for 5 min at temperatures maintained below 4 °C. After centrifugation, 200 µL of the clear supernatant was transferred into a fresh tube containing the purification solution, briefly mixed, and then centrifuged again under the same conditions. From the clarified extract, 175 µL of the upper phase was collected into pre-labelled glass vials and subjected to lyophilization overnight.

#### Chemical derivatization and sample conditioning

Lyophilized residues were derivatized in a two-step process. Initially, 50 µL of the primary derivatizing (methoxamine in pyridine) agent was added and vortexed at 1200 rpm for 90 min at ambient temperature. Following this, 25 µL of the secondary reagent (BSTFA + TMCS) was introduced with an identical vortexing protocol. After derivatization, the solutions were transferred into autosampler-compatible inserts, centrifuged once more (16,000 rpm, 5 min, 4 °C), and prepared for GC–MS analysis.

#### Gas chromatography-mass spectrometry (GC–MS)

##### Instrumentation and analytical conditions

Metabolite separation and detection were performed on a Shimadzu GC-2010 Plus system coupled to a 2010 Plus single quadrupole mass spectrometer (Shimadzu, Kyoto, Japan). A 2 µL aliquot of the derivatized sample was introduced into a CP-Sil 8 CB capillary column (30 m × 0.25 mm internal diameter, 1.00 µm film thickness, Agilent J&W Scientific). Helium was employed as the carrier gas, with flow rates modulated to achieve a linear velocity of 39 cm/s. The oven temperature program began at 100 °C (1-min hold), increased at 6 °C/min to 320 °C, and remained isothermal for an additional 2.33 min. Injections were conducted in split mode (1:5 ratio), using electron impact ionization at 70 eV. Data acquisition was carried out in full scan mode (35–600 m/z) with a scan rate of 3333 amu/sec. A solvent delay of 5 min was applied, with each analytical cycle lasting 40 min.

#### Metabolite annotation and data filtering

##### Identification protocol and quality thresholds

Detected features were matched to spectral entries in the NIST 2014 database (NIST, Gaithersburg, MD, USA). Metabolite identification adhered to the Metabolomics Standards Initiative (MSI) guidelines [21], with compounds retained at Level 2 confidence if their spectral similarity was ≥ 85% and linear retention index deviation ≤ 50. Metabolites detected in fewer than 80% of the samples were excluded from downstream analyses. A comprehensive list of all detected features, including annotated and unannotated metabolites along with their detection prevalence, is provided in Supplementary Table S8. Low-abundance and poorly resolved peaks were discarded. When multiple analytes arose from a single precursor molecule (e.g., carbohydrate derivatives), each was treated as a discrete feature. Where possible, relevant metabolite identification was verified with authentic standards to achieve MSI Level 1 certainty.

#### Data normalization and statistical analysis

##### Univariate and multivariate testing

All statistical analyses were performed using the R statistical environment [22]. The Shapiro–Wilk test assessed the normality of variable distributions. Welch’s ANOVA was used for group comparisons under unequal variance assumptions, followed by Tukey’s HSD for post hoc testing. Statistical significance was defined as *p* < 0.05. To address multiple comparisons in metabolite-level analyses, *p*-values were adjusted using the Benjamini–Hochberg false discovery rate (FDR) method. Adjusted *p*-values (q-values) were calculated for all univariate comparisons and are presented in the Supplementary Tables. Significance thresholds were interpreted based on FDR levels (q < 0.10, q < 0.05, and q < 0.01). Analyses used available-case data at each time point. No imputation was performed for missing metabolomic or clinical variables to maintain data integrity. Variations in sample size across analyses reflect data availability rather than selective inclusion.

To account for potential confounding effects, covariates were selected a priori based on clinical relevance and included age, sex, BMI, and pharmacological treatments. Medication use was evaluated longitudinally and did not significantly differ across time points.

In addition, a residualization approach was applied to metabolomic data, whereby each metabolite feature was adjusted using linear regression models including the selected covariates. Residuals were subsequently used for downstream multivariate analyses to assess the robustness of metabolomic signatures. Full methodological details and implementation are provided in the Supplementary Methods (Sect. S5), with corresponding results reported in Supplementary Figs. S1–S3.

##### Preprocessing and multivariate modelling

Raw chromatograms were aligned using the parametric time warping (PTW) algorithm as implemented by Wehrens et al. [23]. Quantitative data were normalized to the internal standard (2-isopropyl malic acid), log-transformed using a generalized logarithmic transformation to stabilize variance, and autoscaled (mean-centering and division by standard deviation) to ensure comparability across features. Features detected in fewer than 80% of samples were excluded prior to analysis to reduce noise and improve robustness. Missing values were not imputed; all analyses were conducted using available-case data at each time point. This approach was chosen to avoid the introduction of artificial signal in high-dimensional metabolomic data. A detailed description of preprocessing, normalization, and missing-value handling is provided in the Supplementary Methods (Sect. S1).

Classification performance was evaluated using Partial Least Squares Discriminant Analysis (PLS-DA), implemented through the `plsr` function from the R `pls` package. Model validation included permutation testing and k-fold cross-validation with the `caret` package [24]. To correct sample class imbalance, synthetic data augmentation was conducted using the SMOTE algorithm [25].

##### Feature selection and class definition

Variable Importance in Projection (VIP) scores were computed to highlight discriminant metabolites. Cardiac remodeling was evaluated by measuring the left ventricular mass normalized to body surface area (LVMI), derived from standard transthoracic echocardiographic assessments. Subjects were classified according to guideline-recommended sex-specific thresholds for left ventricular hypertrophy: > 115 g/m^2^ for men and > 95 g/m^2^ for women, as defined by the American Society of Echocardiography (ASE) [20] and the European Association of Cardiovascular Imaging (EACVI) [26]. Patients whose LVMI values decreased below the threshold over the course of the intervention were categorized as favorable remodelers, while those with persistently elevated or increased values were considered non-responders. This binary classification was used to support supervised multivariate modeling and stratified analyses of metabolomic profiles. The robustness of class separations was evaluated through permutation testing based on training accuracy and the ratio of between-class to within-class variance.

To characterize longitudinal geometric adaptation, participants were categorized into three RWT trajectory groups based on direction and magnitude of change between baseline and follow-up. Absolute change in RWT (ΔRWT) was calculated for each participant. Individuals were then stratified into three classes:

- Class 0 (stable geometry, n = 42; 26 T0, 16 T4) individuals with RWT below the cut-off at baseline who maintained normal geometry throughout follow-up.
- Class 1 (remodelers, n = 27; 14 T0, 13 T4): individuals with elevated RWT at baseline who improved to below-threshold values;
- Class 2 (non-remodelers, n = 7; 4 T0, 3 T1): individuals with elevated RWT at baseline who exhibited persistently high values over observed time.

Cut-offs for trajectory grouping were defined a priori based on distributional characteristics of ΔRWT to allow balanced subgroup sizes while preserving biological interpretability. This approach was chosen to capture heterogeneity in geometric response rather than relying exclusively on mean cohort changes.

Medication classes were examined descriptively across time points to assess potential confounding effects on remodeling trajectories; however, no significant longitudinal differences were observed, and thus medication variables were not included in multivariate metabolomic models (Supplementary Sect. 5, Supplementary Figs. S1–S3).

#### Pathway enrichment and feature overlap analysis

Metabolic pathway enrichment was performed via MetPa (Metabolomics Pathway Analysis) [27], utilizing curated pathways from the KEGG database ([28]. Common and unique metabolites across class comparisons were represented using UpSet diagrams generated by the UpSetR package in R [29].

#### *Identification of clinical predictors *via* LASSO regression*

To determine metabolite predictors of continuous clinical endpoints—such as changes in LVMI, RWT and VAI—a Least Absolute Shrinkage and Selection Operator (LASSO) regression was employed. The data analysis pipeline was implemented in Python, incorporating preprocessing with MinMax normalization, division into training and testing subsets, and model fitting using cross-validated optimization of the regularization parameter (α). Metabolites with non-zero regression coefficients were interpreted as influential contributors to clinical improvement in hepatic parameters.

## Results

### Study cohort baseline characteristics

At baseline (T0), the study cohort consisted of 127 adults with a mean age of 38.8 ± 11.5 years, and a predominance of females (64.6%). The mean BMI was 42.9 ± 6.0 kg/m^2^, consistent with severe obesity; notably, 63% of participants met the criteria for obesity class III (≥ 40 kg/m^2^). Central adiposity was marked, with a mean waist circumference of 125.1 ± 16.2 cm and hip circumference of 134.1 ± 16.4 cm. (Table 1). Cardiometabolic comorbidities were common: 31.5% of participants were found to have hypertension, all of whom received this diagnosis at the time of enrollment, having no prior history of hypertension. In addition, 22.8% of subjects reported a family history of cardiovascular disease, including coronary artery disease and other major cardiovascular conditions. At baseline, a substantial proportion of patients exhibited cardiometabolic risk factors, including type 2 diabetes mellitus and concentric left ventricular remodeling, providing a clinically relevant framework for subsequent analyses (Table 2).

**Table 1:** Longitudinal changes in clinical parameters over a 24-week follow-up period

Vital parameters	T0 (Baseline)	4 Weeks	12 Weeks	24 Weeks	ANOVA *p*-value	Post hoc significance
Weight (kg)	120.9 ± 22.3	102.9 ± 17.8	100.5 ± 18.8	93.1 ± 18.3	< 0.001	T0 vs 4w ***, T0 vs 12w ***, T0 vs 24w ***
BMI (kg/m^2^)	42.9 ± 6.0	37.7 ± 5.7	35.2 ± 6.0	34.0 ± 6.7	< 0.001	T0 vs 4w ***, T0 vs 12w ***, T0 vs 24w ***, 4w vs 24w *
BSA (m^2^)	2.25 ± 0.26	2.08 ± 0.23	2.09 ± 0.24	2.00 ± 0.23	< 0.001	T0 vs 12w **, T0 vs 24w ***, T0 vs 4w **
Waist Circumference (cm)	125.1 ± 16.2	111.4 ± 12.3	109.8 ± 13.6	106.1 ± 13.8	< 0.001	T0 vs 12w ***, T0 vs 24w ***, T0 vs 4w ***
Hip circumference (cm)	134.1 ± 16.4	123.7 ± 11.2	118.4 ± 14.6	115.8 ± 13.7	< 0.001	T0 vs 12w ***, T0 vs 24w ***, T0 vs 4w **
Systolic blood pressure (mmHg)	126.4 ± 13.0	121.3 ± 12.2	120.5 ± 11.4	119.9 ± 12.9	< 0.01	T0 vs 24w **
Diastolic blood pressure (mmHg)	82.2 ± 8.6	80.4 ± 6.4	78.4 ± 6.1	77.1 ± 6.9	< 0.01	T0 vs 24w **
Heart rate (bpm)	77.1 ± 11.3	68.0 ± 10.8	64.4 ± 8.7	66.1 ± 6.7	< 0.001	T0 vs 12w ***, T0 vs 24w ***, T0 vs 4w ***

Values are presented as mean ± standard deviation (SD). Statistical significance was assessed using repeated-measures ANOVA followed by Tukey’s post hoc test. Asterisks indicate the level of significance compared to baseline (T0): *p* ≤ 0.05 (*), *p* ≤ 0.01 (**), *p* ≤ 0.001 (***), ns = not significant. The analysis highlights significant reductions in body weight, BMI, waist and hip circumferences, as well as improvements in glycemic and lipid profiles and hepatic enzymes. Cardiac function, as measured by ejection fraction and BNP levels, remained stable throughout the study

*BMI* Body mass index; *BSA* Body surface area

**Table 2:** Longitudinal changes in Medical History and medications over a 24-week follow-up period

Disease status	T0 (Baseline)	4 Weeks	12 Weeks	24 Weeks	ANOVA *p*-value
Hypertension, n (%)	18.9	22.5	30.3	24.2	0.90
Elevated blood pressure values, n (%)	18.9	22.5	30.3	24.2	0.96
Dyslipidemia, n (%)	25.2	22.5	24.2	21.2	< 0.05
Type II diabetes mellitus, n (%)	33.9	10	12.1	15.1	0.90
Hyperuricemia, n (%)	3.1	–	–	3.0	0.16
Hypothyroidism, n (%)	18.9	2.5	9.1	9.1	0.96
Smokers, n (%)	11.0	10	12.1	15.1	0.90
Medications					
Statin, n (%)	4.7	7.5	9.1	12.1	0.90
Anti-HTN, n (%)	18.9	22.5	27.3	21.2	0.90
Hypoglycemic, n (%)	2.4	–	–	3.0	0.90
Antiplatelets, n (%)	4.7	5	9.1	9.1	0.90
Thyroid hormone, n (%)	4.7	7.5	9.1	9.1	0.90

Values are presented as mean ± standard deviation (SD). Statistical significance was assessed using repeated-measures ANOVA followed by Tukey’s post hoc test. Asterisks indicate the level of significance compared to baseline (T0): *p* ≤ 0.05 (*), *p* ≤ 0.01 (**), *p* ≤ 0.001 (***), ns = not significant

### Clinical, metabolic, and echocardiographic changes following bariatric surgery

Over the 24-week follow-up period, participants exhibited significant and progressive improvements across a wide range of anthropometric, metabolic, hepatic, and cardiovascular parameters, consistent with the expected effects of structured post-intervention weight reduction (Table 1). The most striking changes were observed in body composition indices. Body weight declined markedly from a baseline value of 120.9 ± 22.3 kg to 102.9 ± 17.8 kg at week 4, 100.5 ± 18.8 kg at week 12, and reached 93.1 ± 18.3 kg by week 24, reflecting a cumulative average reduction of approximately 28 kg. This weight loss was accompanied by a substantial reduction in BMI, from 42.9 ± 6.0 kg/m^2^ at baseline to 34.0 ± 6.7 kg/m^2^ at 24 weeks. Central adiposity, assessed by waist and hip circumferences, also improved significantly, with mean waist circumference decreasing from 125.1 ± 16.2 cm to 106.4 ± 13.4 cm, and hip circumference from 134.1 ± 16.4 cm to 115.8 ± 13.6 cm, indicating a favorable redistribution and reduction of visceral fat mass.

Biochemical markers showed parallel improvements. Fasting glycaemia initially fluctuated at week 4 but demonstrated a sustained reduction, thereafter, culminating in a value of 84.1 ± 19.8 mg/dL at week 24, compared to 95.1 ± 21.0 mg/dL at baseline. Glycated hemoglobin (HbA1c) mirrored this trend, dropping from 5.88 ± 0.51% to 5.61 ± 0.39% by week 12, and remaining stable at week 24, highlighting improved medium-term glycaemia control. Lipid profiles also showed a marked amelioration. Total cholesterol decreased from 180.7 ± 36.2 mg/dL to 171.6 ± 31.1 mg/dL at 24 weeks, while LDL cholesterol levels declined from 107.2 ± 34.1 mg/dL to 100.6 ± 27.4 mg/dL. Conversely, HDL cholesterol increased modestly from 49.4 ± 10.6 mg/dL to 51.6 ± 10.8 mg/dL, and triglyceride concentrations fell by nearly 20 mg/dL, from 105.9 ± 45.1 mg/dL to 85.4 ± 25.5 mg/dL, collectively indicating a beneficial shift in the atherogenic lipid profile. These improvements were also observed among patients with type 2 diabetes mellitus, underscoring the intervention's cardiometabolic impact. Liver enzyme dynamics further supported improved hepatic function. Alanine aminotransferase (ALT) decreased from 38.7 ± 27.5 U/L at baseline to 21.4 ± 8.8 U/L at 24 weeks, while aspartate aminotransferase (AST) declined from 28.1 ± 11.4 U/L to 19.5 ± 4.5 U/L, consistent with regression of hepatic steatosis or subclinical inflammation. Although brain natriuretic peptide (BNP) showed a mild increase at 24 weeks (45.7 ± 27.1 pg/mL), which remained within clinically acceptable limits and may reflect volume redistribution or improved cardiac preload rather than decompensation (Table 3). In fact, the mild but significant increase in BNP at 24 weeks occurred in the context of stable diastolic indices. Obesity is characterized by a “natriuretic peptide recovery” due to the over-expression of natriuretic peptide receptor-C (NPR-C) in adipose tissue, which enhances the clearance of circulating BNP. The reduction in visceral adiposity following surgery diminishes this clearance thereby restoring systemic BNP levels. This interpretation is supported by the stability of our cohort’s diastolic indices and ejection fraction, suggesting that the BNP rise is a marker of favorable metabolic recalibration rather than worsening of cardiac loading conditions [30–32].

**Table 3:** Longitudinal changes biochemical parameters over a 24-week follow-up period

Biochemical parameters	T0 (Baseline)	12 Weeks	24 Weeks	ANOVA *p*-value	Post hoc significance
Fasting Glucose (mg/dL)	95.4 ± 23.6	88.6 ± 12.7	84.1 ± 19.8	0.15	–
HbA1c (%)	5.69 ± 0.87	5.41 ± 0.44	5.52 ± 0.60	0.62	–
Hb (g/L)	13.8 ± 1.5	13.1 ± 0.9	13.5 ± 0.9	0.23	–
PLTS	258.6 ± 65.1	239.6 ± 54.0	256.1 ± 73.5	0.59	–
WBC	8.1 ± 2.7	6.4 ± 1.8	7.0 ± 2.1	0.06	–
Creatinine	0.84 ± 0.20	0.75 ± 0.19	0.81 ± 0.23	0.31	
eGFR	127.0 ± 34.9	97.0 ± 26.5	115.0 ± 0.0	–	–
Urea	30.5 ± 10.1	29.2 ± 8.2	30.6 ± 13.0	0.91	–
Sodium	137.7 ± 2.6	141.7 ± 2.2	141.3 ± 3.6	< 0.001	T0 vs 12w ***, T0 vs 24w ***
Potassium	4.14 ± 0.30	4.14 ± 0.31	4.41 ± 0.55	< 0.05	T0 vs 24w *
Calcium	9.33 ± 0.47	9.77 ± 0.40	9.37 ± 0.50	< 0.01	T0 vs 12w **
Uric Acid	5.87 ± 1.50	5.89 ± 1.26	5.55 ± 1.40	0.74	–
Total Cholesterol (mg/dL)	192.5 ± 37.8	179.8 ± 24.6	183.6 ± 38.9	0.34	–
LDL Cholesterol (mg/dL)	122.1 ± 30.7	108.5 ± 21.7	115.5 ± 35.0	0.23	–
HDL Cholesterol (mg/dL)	50.0 ± 11.3	53.7 ± 16.6	52.2 ± 13.6	0.49	–
Triglycerides (mg/dL)	105.9 ± 45.1	89.4 ± 25.4	85.4 ± 25.5	0.11	–
ALT (U/L)	32.8 ± 19.0	16.7 ± 4.5	15.6 ± 6.9	< 0.05	T0 vs 24w *
AST (U/L)	24.1 ± 9.5	19.2 ± 3.8	15.1 ± 3.2	< 0.05	T0 vs 24w *
Bilirubin	0.70 ± 0.28	0.81 ± 0.33	0.67 ± 0.15	0.75	–
C-reactive protein	0.99 ± 0.92	5.04 ± 6.49	4.81 ± 3.77	< 0.001	T0 vs 12w ***, T0 vs 24w ***
BNP (pg/mL)	19.5 ± 14.3	28.4 ± 8.9	42.2 ± 55.1	< 0.05	T0 vs 24w *

Values are presented as mean ± standard deviation (SD). Statistical significance was assessed using repeated-measures ANOVA followed by Tukey’s post hoc test. Asterisks indicate the level of significance compared to baseline (T0): *p* ≤ 0.05 (*), *p* ≤ 0.01 (**), *p* ≤ 0.001 (***), ns = not significant. The analysis highlights significant reductions in body weight, BMI, waist and hip circumferences, as well as improvements in glycemic and lipid profiles and hepatic enzymes. Cardiac function, as measured by ejection fraction and BNP levels, remained stable throughout the study. The 4-week time point is not included in this longitudinal analysis, as biochemical parameters were not collected on that visit

*HbA1c* Glycated Hemoglobin; *Hb* Hemoglobin; *PLTS* Platelets; *WBC* White blood cells; *ALT* Alanine Aminotransferase; *AST* Aspartate Aminotransferase; *BNP*: B-type Natriuretic Peptide

Echocardiographic parameters showed parallel, favorable changes. RWT demonstrated a statistically significant progressive reduction over time (*p* < 0.001), whereas left ventricular mass index (LVMI) showed a downward numerical trend but did not reach statistical significance at the whole-cohort level (*p* = 0.67). Left ventricular ejection fraction remained preserved throughout follow-up. These findings suggest that early geometric adaptation was driven primarily by changes in RWT to cavity dimension rather than by a uniform reduction in indexed myocardial mass across all participants. Notably, substantial interindividual variability characterized longitudinal echocardiographic trajectories. While RWT decreased significantly at the group level, the magnitude and direction of change varied considerably across participants, and LVMI did not demonstrate a statistically significant mean reduction. These findings indicate that remodeling was not uniform across the cohort and caution against interpreting the observed changes as population-wide structural normalization. Rather, distinct remodeling phenotypes emerged, forming the basis for subsequent metabolomic stratification analyses. Furthermore, no new signs of ventricular dilation, hypertrophy, or diastolic dysfunction were reported in the serial data (Table 4).

**Table 4:** Longitudinal changes in Echocardiographic parameters over a 24-week follow-up period

Parameter	T0 (Baseline)	4 Weeks	12 Weeks	24 Weeks	ANOVA *p*-value	Post hoc significance
Ejection fraction (%)	63.7 ± 5.9	64.0 ± 7.0	63.7 ± 6.0	63.1 ± 5.8	0.94	–
Ascending Aorta (mm)	31.1 ± 3.5	31.2 ± 3.3	30.8 ± 3.1	31.2 ± 3.2	0.97	–
PAPs (mmHg)	18.1 ± 5.4	17.5 ± 6.0	18.7 ± 5.9	18.1 ± 5.5	0.83	–
IVSd (mm)	10.3 ± 1.2	9.75 ± 1.28	9.55 ± 1.06	8.95 ± 1.30	< 0.001	T0 vs 12w *, T0 vs 24w ***, 4w vs 24w *
LVPWd (mm)	8.67 ± 1.13	8.22 ± 1.07	8.12 ± 1.58	7.61 ± 0.86	< 0.001	T0 vs 24w ***
dVStd (mm)	47.8 ± 5.0	47.2 ± 5.6	48.0 ± 5.5	48.1 ± 4.4	0.88	–
RA area (cm^2^)	14.0 ± 3.1	13.2 ± 3.2	14.3 ± 3.4	13.9 ± 3.4	0.51	–
RWT	0.40 ± 0.06	0.39 ± 0.07	0.37 ± 0.06	0.35 ± 0.04	< 0.001	T0 vs 24w ***, 4w vs 24w *
E wave (m/s)	76.8 ± 14.9	75.3 ± 14.2	75.5 ± 16.5	77.3 ± 15.9	0.91	–
A wave (m/s)	67.0 ± 15.4	68.1 ± 16.9	66.6 ± 14.3	60.2 ± 11.4	0.10	–
E/A	1.20 ± 0.35	1.16 ± 0.33	1.17 ± 0.31	1.32 ± 0.34	0.17	–
Deceleration time (ms)	209.9 ± 42.2	223.1 ± 49.8	215.7 ± 53.0	202.3 ± 29.6	0.19	–
E/e’	6.68 ± 1.40	6.75 ± 1.23	6.26 ± 1.54	6.53 ± 0.99	0.37	–
LAVi (ml/m^2^)	23.0 ± 6.1	23.5 ± 6.6	25.0 ± 7.7	24.7 ± 6.4	0.32	–
TAPSE (mm)	24.9 ± 3.0	24.1 ± 3.3	24.9 ± 3.2	24.7 ± 3.8	0.53	–
RVs’ (cm/s)	13.7 ± 2.4	13.0 ± 2.3	13.2 ± 2.0	12.8 ± 2.0	0.09	–
LVEDV (mL)	106.4 ± 30.7	101.9 ± 28.8	113.4 ± 36.3	102.4 ± 28.2	0.39	–
LVESV (mL)	39.1 ± 14.4	37.4 ± 14.8	41.7 ± 16.2	38.4 ± 13.5	0.64	–
E’l wave (cm/s)	13.2 ± 3.4	13.3 ± 3.5	14.3 ± 3.7	13.8 ± 2.9	0.40	–
E’s wave (cm/s)	10.4 ± 2.9	9.57 ± 2.43	10.5 ± 2.9	10.1 ± 2.5	0.34	–
RVd1 (mm)	31.3 ± 4.1	30.1 ± 5.9	31.1 ± 3.8	29.5 ± 4.2	0.13	–
LVMI (g/m^2^)	71.9 ± 13.8	70.8 ± 13.5	70.6 ± 12.0	68.7 ± 13.5	0.67	–

Values are presented as mean ± standard deviation (SD). Statistical significance was assessed using repeated-measures ANOVA followed by Tukey’s post hoc test. Asterisks indicate the level of significance compared to baseline (T0): *p* ≤ 0.05 (*), *p* ≤ 0.01 (**), *p* ≤ 0.001 (***), ns = not significant

*PAPs* Pulmonary Artery systolic Pressure; *IVSd* Interventricular Septum at end-diastole; *LVPWd* Left Ventricular Posterior Wall at end-diastole; *dVSTd* End-diastolic left Ventricular diameter (Standard view); *RA area*: Right Atrial area; *RWT* Relative wall thickness; *E wave*: peak Early diastolic mitral inflow velocity; *A wave*: peak Late (Atrial) diastolic mitral inflow velocity; *E/A*: Ratio of Early to Late diastolic mitral inflow velocity; *E/e’*: ratio of mitral inflow Early diastolic velocity to mitral Annular Early diastolic tissue velocity; *LAVi*: indexed Left Atrial Volume; *TAPSE*: Tricuspid Annular Plane Systolic Excursion; *RVs’*: Right Ventricular systolic excursion velocity; *LVEDV*: Left ventricular End-Diastolic Volume; *LVESV*: Left Ventricular End-Systolic Volume; E’l wave: Early diastolic velocity at the lateral mitral annulus; E’s wave: Early diastolic velocity at the septal mitral annulus; *RVd1*: Right Ventricular basal diameter; *LVMI*: left ventricular mass index

It is noteworthy that modest variations in pharmacological therapy were observed during follow-up, particularly in the use of antihypertensive agents and statins (Table 2). However, the proportion of patients receiving these medications remained relatively stable over time, without statistically significant longitudinal differences. Moreover, the early structural changes in RWT and LVMI were observed within the first 4–12 weeks, a timeframe during which medication adjustments were limited. These observations reduce the likelihood that pharmacological modifications alone account for the early remodeling patterns detected in this cohort. To further assess the potential impact of confounding variables, additional analyses were performed on residualized metabolomic data. These analyses confirmed that group separation and model performance were preserved after adjustment, indicating that the observed metabolic signatures are not primarily driven by clinical covariates (see Supplementary Figs. S1–S3).

### Early left ventricular remodeling after bariatric surgery

**Fig. 2:**
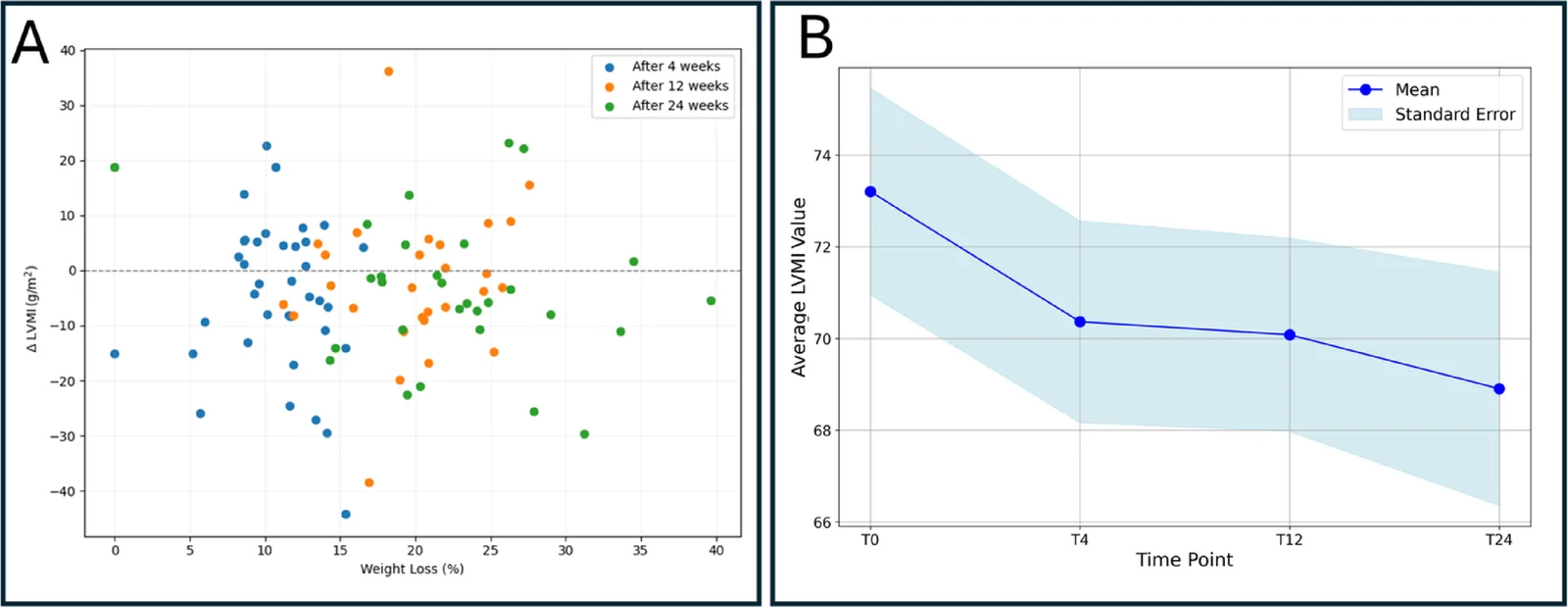
Early cardiac remodeling response. **A** Scatter plot showing change in LVMI ratio as a function of percent weight loss at 4, 12, and 24 weeks. Two response phenotypes are observed: patients with improved remodeling (ΔLVMI < 0) and those with worsening (ΔLVMI > 0), independent of weight loss. **B** Longitudinal trajectory of mean LVMI values over time, showing a steeper early decline between baseline and 4 weeks, followed by a more gradual trajectory thereafter

Echocardiographic assessment revealed that early geometric changes were detectable after bariatric surgery, with the most pronounced adaptations occurring within the first 4 to 12 weeks. To investigate the relationship between weight loss and cardiac remodeling, we evaluated changes in the left ventricular mass normalized to body surface area (LVMI) as a function of percentage weight loss at 4-, 12-, and 24-weeks post-intervention (Fig. 2A). Despite a general trend toward improved remodeling with increasing weight reduction, we observed substantial interindividual variability. Notably, patients stratified into two distinct subgroups—those who experienced a reduction in LVMI and those in whom the ratio increased, independent of the amount of weight lost.

Temporal analysis of LVMI (Fig. 2B) showed a steeper early decline between baseline and 4 weeks, followed by a more gradual trajectory thereafter; however, these changes did not reach statistical significance.

To formally evaluate the relationship between weight loss and cardiac remodeling, we performed a multivariable linear regression analysis including ΔBMI, baseline LVMI, age, and sex as covariates. In this model, ΔBMI was not significantly linked to changes in LVMI (β = − 1.31, *p* = 0.29), while baseline LVMI proved to be a strong predictor of remodeling (β = − 0.55, *p* < 0.001). Detailed regression results are available in Supplementary Table S9 and Fig. S4. These results show that the amount of weight loss alone does not account for the variability in cardiac remodeling response observed in this group. They further support the idea that cardiac remodeling after bariatric surgery is influenced by factors beyond just weight loss, including baseline structural status and metabolic adaptation.

To better visualize this heterogeneity at the individual level, we performed additional longitudinal analyses based on both continuous and categorical measures of cardiac geometry. Spaghetti plots of RWT showed significant variation between individuals in early structural adaptation, while alluvial diagrams highlighted dynamic shifts across the clinical threshold for concentric remodeling (Supplementary Fig. S5). These methods emphasize that early structural changes vary and follow different paths across patients. Longitudinal visualization of metabolomic changes was further examined using PLS-DA score projections based on median-normalized, log-transformed, and autoscaled data. Individual trajectories and group trends were shown in a reduced latent variable space (Supplementary Fig. S6).

However, despite a shared overall trend toward reverse remodeling, substantial interindividual variability was evident. While a subset of patients demonstrated an early and marked regression of concentric geometry, others exhibited a more modest or delayed structural response. This temporal pattern suggests that early remodeling signals can be detected shortly after surgery, whereas the full differentiation of remodeling trajectories becomes more evident over time. These findings highlight heterogeneity in cardiac remodeling trajectories after bariatric surgery and underscore the need to explore factors potentially associated with differential structural adaptation.

**Fig. 3:**
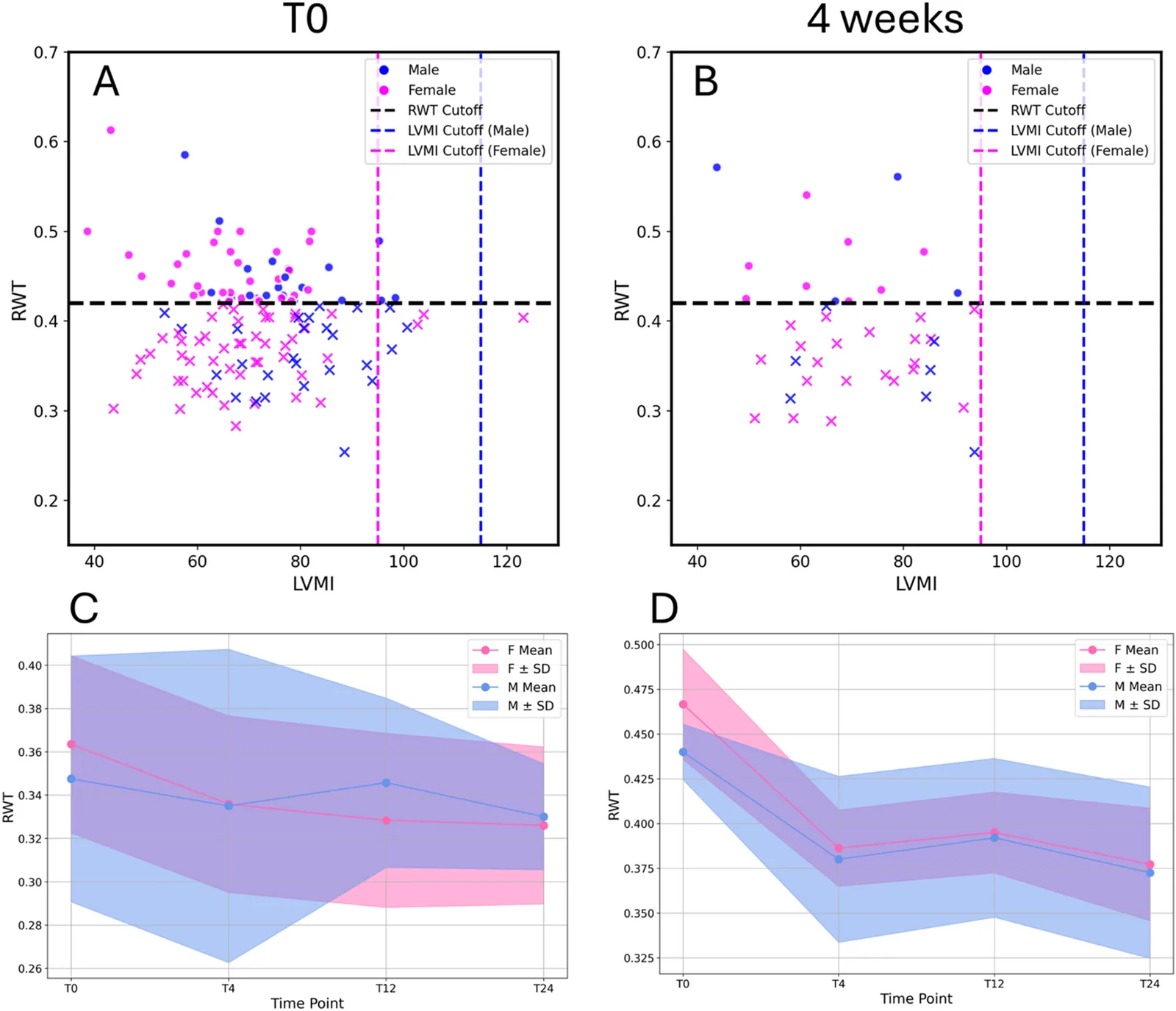
Longitudinal assessment of left ventricular geometry and remodeling patterns after bariatric surgery. **A** and **B** Scatter plots showing the relationship between LVMI and RWT at baseline (T0) and 4 weeks after surgery (T4). Each dot represents an individual participant (blue = male, pink = female). Dashed horizontal and vertical lines indicate diagnostic cutoffs for concentric remodeling (RWT = 0.42) and LV hypertrophy (LVMI > 115 g/m^2^ for males, > 95 g/m^2^ for females). A marked reduction in the proportion of subjects exceeding the RWT threshold was observed from T0 to T4, consistent with early reversal of concentric geometry. **C** and **D** Longitudinal trends of mean RWT (± SD) over 24 weeks of follow-up in females (pink) and males (blue). Shaded areas represent standard deviation bands. Panels illustrate differential remodeling trajectories, with progressive normalization of cardiac geometry over time and a more pronounced early improvement in women

To evaluate this hypothesis, we divided a subset of patients into distinct groups based on their cardiac geometry recovery assessed at 4 weeks after intervention. As shown in Fig. 3 at T0, 24 out of 50 patients (48.0%) had an RWT above the diagnostic threshold, indicating concentric remodeling. This prevalence significantly decreased at 4 weeks (12 out of 38 patients; 31.5%).

Based on early changes in RWT at 4 weeks and subsequent longitudinal trajectories, patients were stratified into three remodeling classes: Class 0, individuals with RWT below the cut-off at baseline who maintained normal geometry throughout follow-up (17 women and 9 men); Class 1, individuals with elevated RWT at baseline who rapidly improved to below-threshold values during 4 weeks follow-up (9 women and 5 man); and Class 2, individuals with elevated RWT at baseline who exhibited a late-response with persistently high values over observed time. This classification enabled detailed monitoring of longitudinal geometry changes and facilitated the identification of distinct remodeling phenotypes. Once again, this classification should be interpreted as exploratory and intended to capture initial geometric trajectories rather than definitive remodeling outcomes.

### Systemic metabolic profiles associated with cardiac remodeling trajectories

To explore whether heterogeneity in cardiac remodeling was associated with systemic metabolic differences, supervised multivariate analyses were applied to untargeted metabolomic data. Partial least squares–discriminant analysis (PLS-DA) demonstrated a clear separation between patients exhibiting early left ventricular reverse remodeling and those with a blunted structural response.

**Fig. 4:**
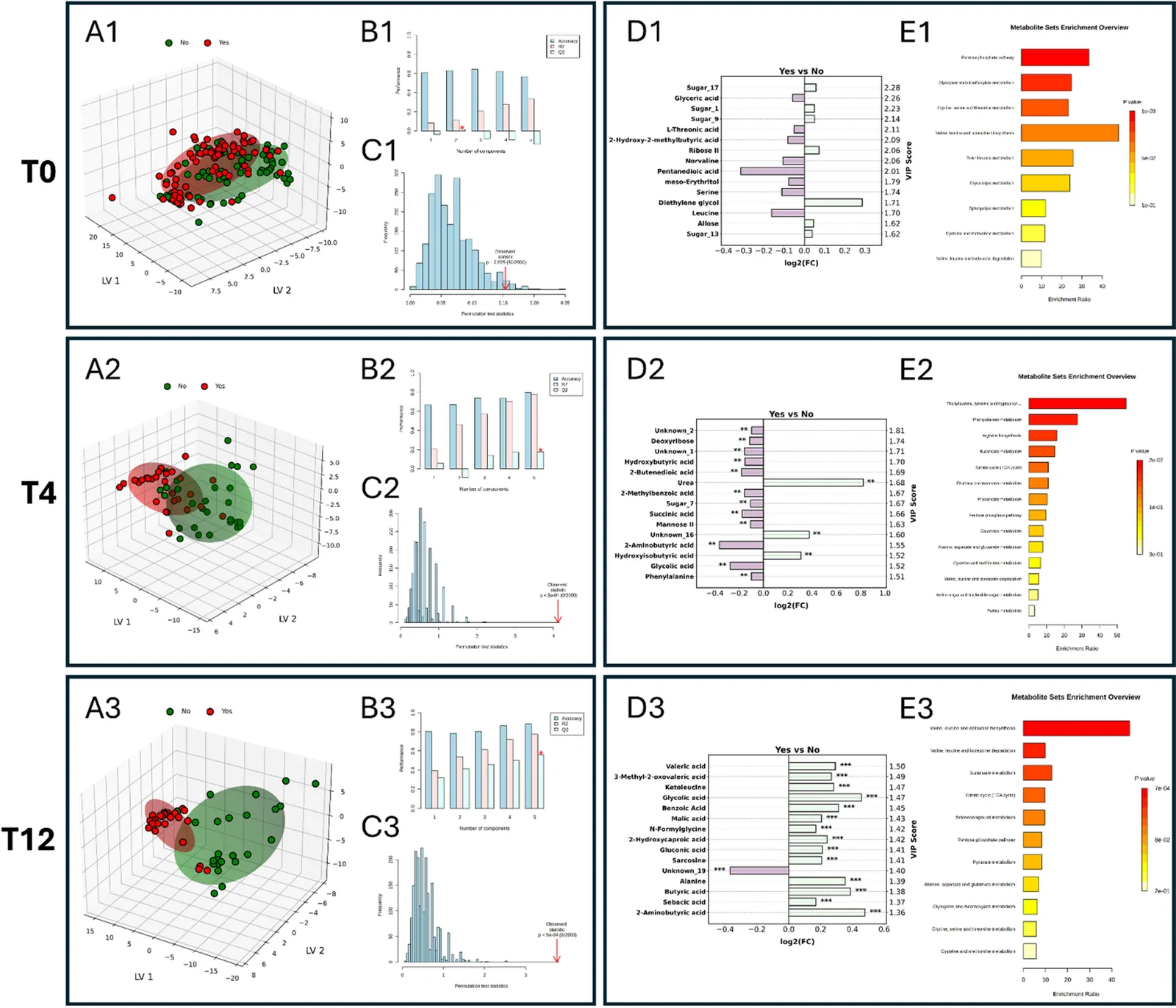
Metabolomic separation of patients with and without cardiac remodeling across post-intervention timepoints. **A1**–**A3** PLS-DA score plots showing the distribution of metabolic profiles between patients without (green) and with (red) concentric cardiac remodeling at 0, 4, and 12 weeks, respectively. Ellipsoids represent 95% confidence intervals. **B1**–**B3** Cross-validation performance plots demonstrating model stability across timepoints. **C1**–**C3** Permutation tests (n = 1000) confirming that classification performance was not due to chance. **D1**–**D3** Bar plots of log2 fold-change for metabolites with VIP score > 1.5 contributing to class separation. Asterisks denote statistical significance based on FDR-adjusted q-values (*q < 0.10, **q < 0.05, ***q < 0.01). **E1**–**E3** Metabolite Set Enrichment Analysis (MSEA) showing enriched metabolic pathways distinguishing responders from non-responders at each timepoint, ranked by enrichment ratio and adjusted p-values. Complete univariate statistics, including nominal p-values and FDR-adjusted q-values, are provided in Supplementary Tables S1–S3

Given the ability of bariatric surgery to induce a metabolic remodeling, we then employed an untargeted metabolomic analysis to characterize systemic metabolic differences between individuals with and without evidence of cardiac remodeling following the intervention. As shown in Fig. 4, supervised PLS-DA models were constructed at each time point (0, 4, and 12 weeks) to discriminate between participants with (red) or without (green) cardiac remodeling. The multivariate score plots (Fig. 4, panels A1–A3) revealed that before the surgery (A1), metabolic differences between the two groups were still modest, as reflected by partial overlap in the PLS-DA space. However, by 4 (A2) and especially 12 weeks (A3), a more precise separation emerged, suggesting that the degree of cardiac remodeling is better mirrored by progressively distinct metabolic signatures as weight loss advances.

Model validation through cross-validation (Fig. 4, panels B1–B3) and permutation testing (Fig. 4, panels C1–C3) offered a detailed view of the model's performance. At baseline (T0), classification accuracy was modest (accuracy = 0.60, R^2^ = 0.15, Q^2^ = 0.03), with some overlap between groups, although permutation testing confirmed statistical significance (p = 0.04). Notably, the model's performance improved significantly at post-intervention timepoints. At 4 weeks (T4), predictive ability increased considerably (accuracy = 0.80, R^2^ = 0.65, Q^2^ = 0.45; permutation test *p* = 0.001), and this trend continued at 12 weeks (T12), where the model showed higher explanatory and predictive performance (accuracy = 0.80, R^2^ = 0.75, Q^2^ = 0.50; *p* = 0.001). These results suggest that metabolomic separation becomes increasingly stronger over time after bariatric surgery. Similar validation results were obtained after adjusting for clinical confounders, further confirming the robustness of the identified metabolomic patterns (Supplementary Figs. S1–S3).

The metabolites driving this separation (VIP > 1.5) are reported in the fold-change bar plots (Fig. 4 panels D1–D3). Complete univariate statistics, including nominal *p*-values and FDR-adjusted q-values for all metabolites across time points, are reported in Supplementary Tables S1–S3. At enrollment (D1), early differences were observed in the levels of several sugars, including ribose and allose, in the remodeled group, while glyceric, threonic, 2-hydroxy-2-methylbutyric, and pentanedioic acids, as well as norvaline, meso-erythritol, serine, and leucine, were lower. At 4 weeks (D2), discriminatory metabolites included decreased levels of deoxyribose, 2-butanedioic, hydroxybutyric, 2-methylbenzoic, succinic, aminobutyric, and glycolic acids as well as mannose and phenylalanine. In contrast, urea and hydroxyisobutyric acid increase. By 12 weeks (D3), individuals with cardiac remodeling showed clear enrichment in short-chain fatty acids (valeric and butyric), glycolic, benzoic, malic, 2-hydroxycaproic, gluconic, and sebacic acids and intermediates of branched-chain amino acid metabolism (3-methyl-2oxovaleric acid, ketoleucine). The observed metabolic profile is consistent with alterations in pathways related to gut-derived metabolites and mitochondrial metabolism. Specifically, the accumulation of malic acid may indicate reduced activity of key enzymes in the Krebs cycle, leading to impaired cellular respiration and decreased ATP synthesis. Benzoic acid, in turn, is known for its pro-oxidant effects, which can further compromise mitochondrial efficiency and induce oxidative stress [33].

Consistent with these findings, pathway enrichment analysis (Fig. 4, panels E1–E3) highlighted increasingly specific alterations in pathways related to fatty acid degradation, ketone body metabolism, dicarboxylate processing, and amino acid catabolism in subjects with cardiac remodeling. Detailed pathway enrichment results are provided in Supplementary Table S4.

In complementary exploratory analyses, patients were categorized into remodeling classes based on longitudinal echocardiographic trajectories. These classes differed in both baseline and early postoperative metabolic profiles, again at the level of metabolic pathways rather than individual metabolites.

**Fig. 5:**
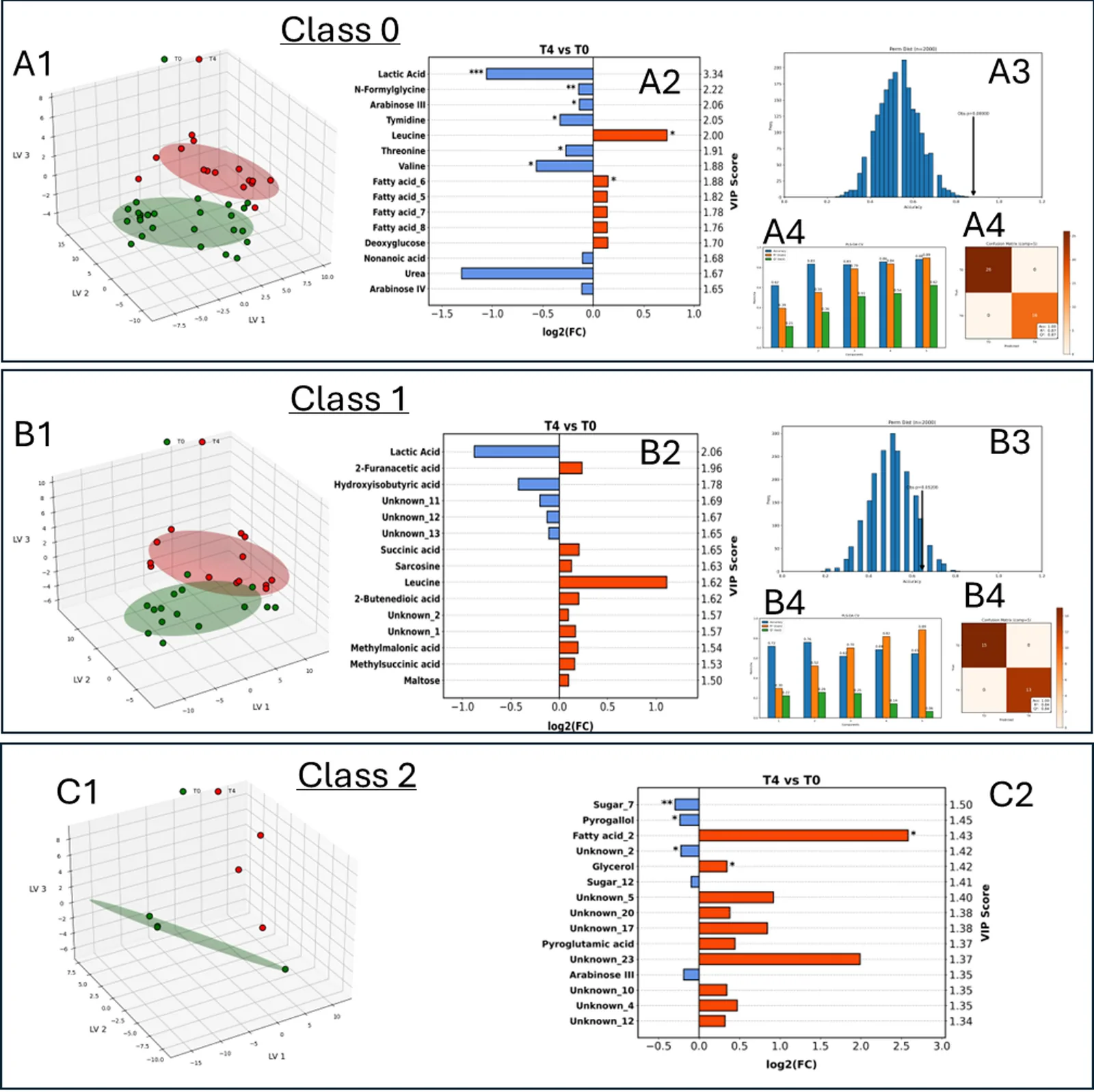
Class-specific metabolomic remodeling patterns from baseline (T0) to 4 weeks (T4) post-bariatric surgery. **A1**–**C1** PLS-DA score plots showing separation between T0 and T4 within each remodeling class: Class 0 (**A1**), Class 1 (**B1**), and Class 2 (**C1**). **A2**–**C2** Log2 fold-change bar plots for metabolites with VIP > 1.5, highlighting decreases (blue) and increases (orange) between T4 and T0. Statistical significance is indicated based on FDR-adjusted q-values (*q < 0.10, **q < 0.05, ***q < 0.01). **A3**–**B3** Permutation test distributions validating model significance. **A4**–**B4** Cross-validation accuracy plots and confusion matrices. Class 0 showed reductions in glycolytic and amino acid metabolites with increased fatty acid turnover; Class 1 exhibited enhanced oxidative metabolism and TCA cycle intermediates; Class 2 demonstrated persistent accumulation of sugars and incomplete metabolic adaptation. Detailed univariate statistics for all metabolites are reported in Supplementary Tables S5–S6

Although LVMI did not show a statistically significant change at 4 weeks, we applied untargeted metabolomics to the three previously identified classes to evaluate potential specific biochemical adaptations from baseline (T0) to 4 weeks (T4) post-surgery (Fig. 5).

In Class 0 (patients with persistently normal RWT), PLS-DA models (A1) showed clear separation between T0 and T4 profiles, supported by robust permutation test statistics (A3) and strong cross-validation accuracy (A4). The most discriminant metabolites (A2) included significant reductions in lactic acid, N-formylglycine, thymidine, threonine, and multiple fatty acid species, alongside increases in arabinose III and leucine. Pathway analysis suggested early shifts in carbohydrate utilization, amino acid turnover, and β-oxidation activity. Given the absence of changes in RWT in this class, the observed effects can be primarily attributed to the weight loss induced by the surgical procedure. Full lists of discriminant metabolites, including fold changes, nominal p-values, and FDR-adjusted q-values for each class comparison, are reported in Supplementary Tables S5–S6.

Class 1 (patients with baseline concentric remodeling who normalized RWT) exhibited a metabolic profile (B1) characterized by reductions in lactic acid, 2-furanoic acid, hydroxyisobutyric acid, succinic acid, and several unidentified metabolites, paralleled by increases in sarcosine, 2-butenedioic acid, methylmalonic acid, and maltose (B2) which points to improved mitochondrial oxidative function, enhanced amino acid catabolism, and increased flux through the TCA cycle. Model validations (B3, B4) are consistent with strong separation and predictive reliability.

By contrast, Class 2 (patients with late response to concentric remodeling improvement) displayed limited separation between T0 and T4 in PLS-DA space (C1), with metabolic shifts dominated by elevations in sugars (e.g., sugar_7, sugar_12), polyphenolic derivatives (pyrogallol), glycerol, and various unannotated metabolites (C2). These changes suggested sustained dysregulation in carbohydrate metabolism and incomplete mitochondrial adaptation, with patients with a higher LMI/BSA ratio showing increased fatty acid levels, which can be attributed to impaired mitochondrial function, reflecting reduced fatty acid oxidation efficiency.

While these findings should be interpreted with caution, given the small sample sizes of these subgroups, especially Class 1, they suggest that early post-surgical metabolic reprogramming is most pronounced in Class 1, aligning with structural recovery. In contrast, Class 2 shows a muted or maladaptive biochemical response despite weight loss.

### Baseline and early metabolic signatures associated with cardiac remodeling response

To investigate whether metabolic differences were already present before surgery, baseline metabolomic profiles were compared between patients according to subsequent cardiac remodeling response. Baseline PLS-DA models demonstrated partial separation between remodeling groups, indicating that pre-intervention metabolic patterns were associated with later structural adaptation.

**Fig. 6:**
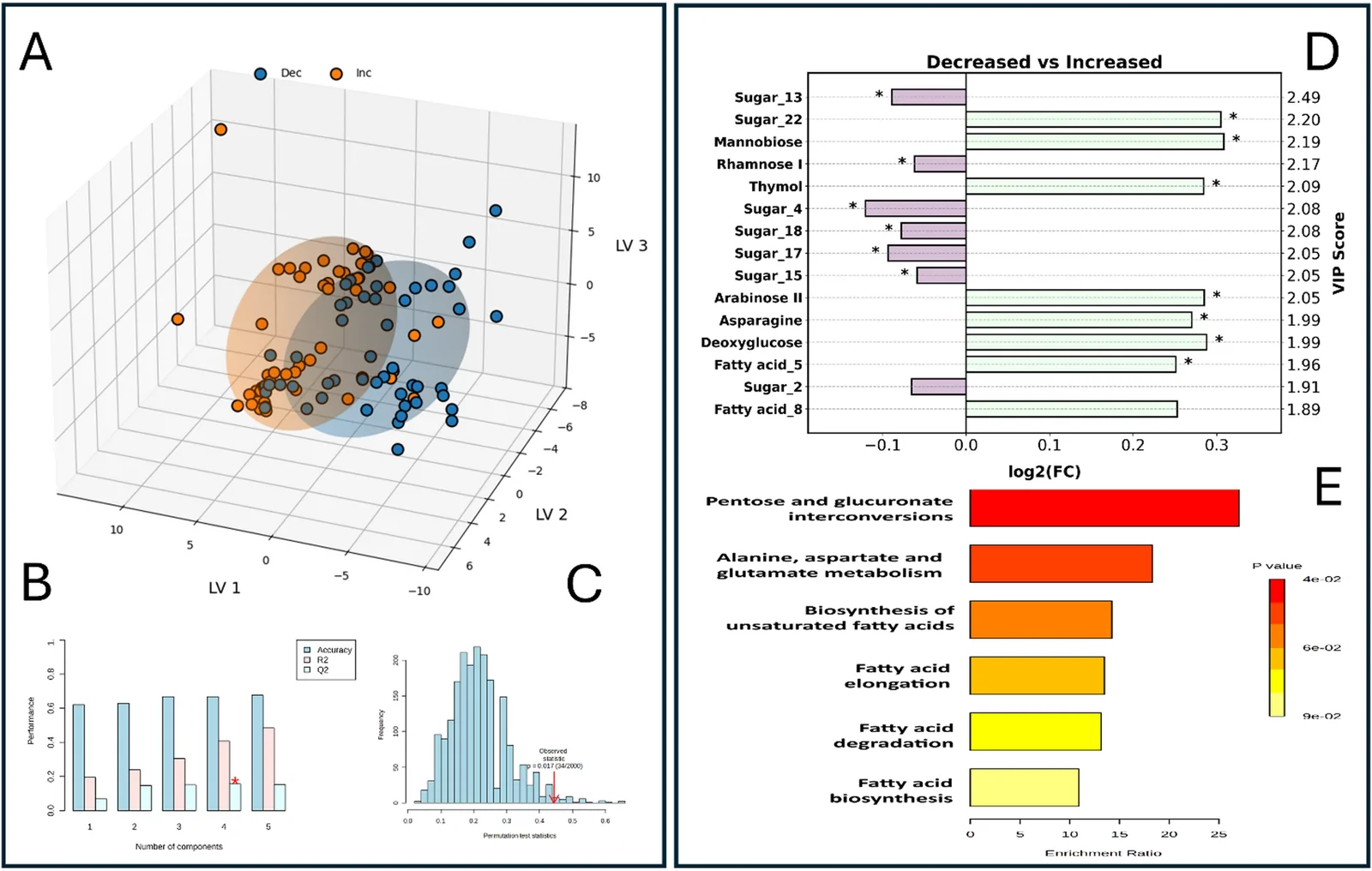
Baseline metabolomic predictors of early cardiac remodeling response. **A** PLS-DA score plot based on baseline metabolomic data, showing discrimination between patients with decreased (orange) vs increased (blue) LVMI following intervention. **B** Model performance assessed by cross-validation. **C** Permutation test confirming model validity. **D** Bar plot of log2 fold-change for baseline metabolites with VIP > 1.5 distinguishing patients with improved vs worsened remodeling trajectories. Statistical significance is indicated based on FDR-adjusted q-values (*q < 0.10, **q < 0.05, ***q < 0.01). **E** Pathway enrichment analysis of discriminating metabolites, highlighting involvement of sugar metabolism, amino acid pathways, and lipid biosynthesis. Complete univariate results, including FDR-adjusted q-values, are reported in Supplementary Table S7

The resulting 3D PLS-DA score plot (Fig. 6A) shows clear separation between patients with decreasing (blue) versus increasing (orange) LVMI, suggesting that baseline metabolic fingerprints are predictive of future cardiac remodeling. Model performance was confirmed by cross-validation (Fig. 6B) and permutation testing (Fig. 6C), both indicating robust discriminatory capacity.

The metabolites most responsible for group separation (VIP > 1.5) are shown in the fold-change plot (Fig. 6D). Corresponding univariate statistics with FDR-adjusted q-values are reported in Supplementary Table S7. Patients who exhibited favorable remodeling trajectories were characterized by elevated baseline levels of sugar-related metabolites (sugar_22, mannobiose, arabinose, deoxyglucose) as well as thymol, asparagine, and several fatty acids, reflecting a metabolic state dominated by anaerobic pathways. In contrast, reduced levels of rhamnose and other poorly annotated sugars (sugar_13, sugar_4, sugar_17, sugar_17, sugar_17, sugar_15, sugar_2) were observed. These differences were functionally linked to pathways involved in pentose and glucuronate interconversions, alanine/aspartate metabolism, and fatty acid biosynthesis and degradation, as highlighted by enrichment analysis (Fig. 6E). Overall, these findings suggest that metabolic predisposition may influence an individual's early remodeling response, regardless of weight loss, and could inform stratification strategies for personalized intervention.

Finally, as an exploratory approach, LASSO regression was applied to identify metabolic features associated with continuous indices of cardiac geometry, including LVMI and RWT. Selected metabolites predominantly mapped to pathways involved in energy metabolism, amino acid turnover, and lipid handling.

The results are presented in Figures S7, predictive features included nonanoic acid, meso-erythritol, 2-furanacetic acid, and mannose I, along with multiple unknown or unannotated sugar species. These findings suggest a remodeling phenotype that may involve altered carbohydrate and organic acid metabolism, potentially linked to intracellular osmotic balance and myocardial stiffness. Given the observational nature of the study, these findings should be interpreted as associative and hypothesis-generating.

## Discussion

Bariatric surgery is increasingly recognized not only as a treatment for obesity and its metabolic complications but also as a potent intervention for reversing adverse cardiac remodeling [34, 35]. At the myocardial level, it can act as a metabolic reset, promoting aerobic metabolism and reducing oxidative stress and fibrotic signaling, thereby facilitating the regression of LVH [36, 37].

Our prospective, longitudinal study shows that obese subjects undergoing bariatric surgery display diverse cardiac remodeling responses, with early, beneficial remodeling linked to specific metabolomic profiles compared to delayed or diminished structural adaptation.

The novelty of our study is situated within the translational landscape by examining how weight-loss-associated changes in serum metabolomic profiles accompany the reversal of myocardial remodeling. Integrating metabolomics with echocardiographic and clinical data provides a unique systems biology approach to deciphering the biochemical signals that may drive cardiovascular recovery [16].

Importantly, the metabolomic profiles analyzed in this study derive from circulating serum metabolites and therefore reflect systemic metabolic states rather than myocardial-specific processes. As such, the observed associations should not be interpreted as direct evidence of cardiac metabolic mechanisms but rather as systemic correlates of structural adaptation.

The early reduction in RWT and the directional, though not statistically significant, decrease in LVMI observed in our cohort, aligns with previous reports of rapid cardiac structural adaptation after substantial weight loss [38, 39]. While RWT decreased significantly over follow-up, the reduction in LVMI did not reach statistical significance when analyzed at the whole-cohort level. Notably, these changes were most pronounced within the first 12 weeks, with a plateau thereafter, highlighting a narrow window during which myocardial remodeling is most responsive. This temporal pattern aligns with the results from the FatWest study, where left ventricular mass and global longitudinal strain improved primarily within the first postoperative year [40]. Similarly, data from Frea et al. [3] indicated a marked regression of LVH and normalization of myocardial strain within 10 months of surgery. Nonetheless, our population consisted predominantly of young individuals with either normal geometry or concentric remodeling at baseline; mean LVMI values were largely within guideline-defined normal limits. Consequently, the potential for detecting large absolute reductions in indexed mass was inherently limited.

Second, concentric remodeling in obesity often begins as an increase in RWT without a significant rise in total myocardial mass. In such cases, RWT may serve as a more sensitive indicator of early geometric changes than LVMI. The notable decrease in RWT thus indicates beneficial remodeling of ventricular shape, even if there are no major changes in total indexed mass.

Third, significant interindividual variability was observed. Subgroup analyses indicate that patients classified as early remodelers showed directional decreases in LVMI, while others did not. When averaging across the entire cohort, these different trajectories likely diminished statistical significance. Importantly, our metabolomic analyses were based on remodeling phenotypes and trajectories rather than just mean cohort changes, supporting the idea that metabolic signatures relate to structural heterogeneity rather than uniform mass regression.

Taken together, these findings suggest that early cardiac adaptation after bariatric surgery may initially involve geometric normalization (a reduction in RWT) before a measurable decrease in global myocardial mass becomes statistically detectable. A longer follow-up might be needed to observe more significant reductions in LVMI.

Although early geometric changes were observed at 4 weeks, the role of hemodynamic unloading should also be considered. Bariatric surgery causes rapid drops in plasma volume and preload, which could influence load-dependent echocardiographic measures. However, in our group, traditional diastolic load-dependent indices (E/A ratio, E/e′, estimated pulmonary artery systolic pressure) and circulating BNP levels showed no signs of acute preload reduction. In fact, BNP remained within normal ranges. While small-volume-related effects may still play a role, the absence of accompanying hormonal or filling-pressure changes suggests that hemodynamics alone do not explain the findings. We interpret the early results as reflecting initial geometric adaptation amid a complex interplay of load reduction and metabolic changes, rather than as clear regression of myocardial hypertrophy. Therefore, although the term remodeling should be used with caution at the 4-week time point, the overall pattern of structural changes across serial assessments supports a progressive rather than purely load-mediated process.

To incorporate these observations into a unified biological framework, our findings support a model where regression of concentric remodeling indicates the recovery of systemic and possibly myocardial metabolic flexibility. Concentric geometry in obesity is metabolically expensive: increased wall thickness raises oxygen demand, while mitochondrial inefficiency and substrate inflexibility limit ATP production. In these conditions, the accumulation of intermediary metabolites—including branched-chain amino acids (BCAAs), dicarboxylic acids, and glycolytic byproducts—may indicate incomplete oxidative flux and redox imbalance, which further favors hypertrophic remodeling. The early reduction in RWT observed in a subset of patients aligned with metabolomic signatures indicating enhanced mitochondrial oxidative capacity: lower lactate and glycolytic intermediates, changes in TCA cycle–related metabolites (such as malate and succinate), and fluctuations in fatty acid and ketone body metabolism. These changes collectively point to improved coupling between substrate availability and oxidative phosphorylation. Instead of merely reflecting caloric restriction, this pattern suggests reactivation of metabolic flexibility—defined as the ability to properly switch between lipid and carbohydrate substrates in response to energy demands [5, 41].

Importantly, patients with blunted or delayed remodeling exhibited persistence of carbohydrate-derived metabolites and incomplete normalization of fatty acid species, a profile compatible with residual mitochondrial inefficiency or impaired β-oxidation flux. This divergence occurred despite comparable weight reduction, reinforcing the concept that myocardial structural adaptation depends not only on mechanical unloading but also on biochemical remodeling.

Thus, our data suggest that bariatric surgery is associated with systemic metabolic reprogramming; however, the extent to which these changes contribute to myocardial structural adaptation cannot be directly established from the present data. In this context, metabolomic signatures may represent systemic correlates—or possibly drivers—of myocardial energetic recovery.

The consistent modulation of branched-chain amino acids (BCAAs) is particularly informative mechanistically. Elevated circulating BCAAs have been linked to insulin resistance, mTOR activation, and maladaptive cardiac concentric remodeling. Impaired myocardial BCAA catabolism results in the buildup of toxic intermediates that disrupt mitochondrial function and increase oxidative stress. The normalization of BCAA-related metabolites seen in early remodelers suggests a restoration of amino acid oxidative pathways and a reduction in metabolic signaling cascades that drive concentric remodeling.

Similarly, the emergence of short-chain fatty acids (SCFAs), including butyrate and valerate, may indicate not only changes in gut microbial composition but also increased systemic metabolic communication [42]. SCFAs are known to enhance mitochondrial efficiency, decrease inflammation, and regulate histone deacetylase activity, thereby affecting cardiac gene expression and substrate use. Their abundance in early remodeling phenotypes supports a model where gut–metabolic–cardiac axis interactions play a role in structural recovery.

Together, these findings align with the hypothesis that restoration of mitochondrial substrate flexibility and redox balance is a central determinant of reverse concentric remodeling.

Other notable changes included declines in nucleotide metabolites such as thymidine and N-formylglycine, suggesting reduced oxidative stress and DNA repair activity, and increased levels of glycine and serine, indicative of restored redox balance, one-carbon metabolism, glutathione cycling, and redox homeostasis.

The LASSO regression model identified metabolite subsets that predicted changes in LVMI and RWT. For LVMI, key predictors included ribose, 5-oxoproline, heptanoic acid, and diethylene glycol—metabolites involved in oxidative metabolism and detoxification. Heptanoic acid’s role in medium-chain β-oxidation may reflect enhanced myocardial fuel efficiency. RWT-associated features, such as nonanoic acid, mannose, and furanacetic acid, point to altered carbohydrate metabolism and the regulation of myocardial stiffness. These observations suggest that osmotic and metabolic regulators of intracellular homeostasis may partially modulate cardiac geometric remodeling.

Unsupervised PCA and PLS-DA analyses revealed that preoperative metabolomic profiles could differentiate patients likely to exhibit favorable cardiac remodeling.

Baseline enrichment in sugars such as mannobiose, arabinose, and deoxyglucose, as well as in asparagine and fatty acids, was predictive of rapid post-intervention LVMI improvement. These patterns suggest pre-existing metabolic resilience, characterized by an intestinal microbiota capable of producing mannose and galactose, a partially efficient tricarboxylic acid cycle that produces oxaloacetate for asparagine synthesis, and incomplete β-oxidation, leading to increased circulating fatty acids. Such substrate flexibility may support enhanced myocardial adaptability.

Conversely, higher levels of rhamnose and unidentified sugar species in patients with a more persistent cardiac remodeling and a late response may denote impaired glycemic regulation or increased intestinal permeability, potentially due to gut dysbiosis [43].

An important consideration when interpreting both the metabolomic and echocardiographic findings is the potential influence of concomitant pharmacological therapy. Consistently, analyses performed after adjustment for major clinical confounders yielded highly comparable results, reinforcing that the identified metabolomic signatures are not primarily driven by demographic or treatment-related factors (Supplementary Figs. S1–S3). Notably, in our cohort, medication use did not change significantly during the observation period, and the magnitude of early remodeling exceeded what would typically be expected from minor therapeutic adjustments alone. Furthermore, the metabolomic signatures associated with early reverse remodeling were enriched for pathways related to mitochondrial substrate utilization, branched-chain amino acid oxidation, short-chain fatty acid metabolism, and TCA cycle intermediates—biochemical domains not classically driven by antihypertensive or statin therapy. While we cannot exclude a modulatory contribution of pharmacological agents, the temporal concordance between weight-loss–associated metabolic reprogramming and structural adaptation supports a predominant role for systemic metabolic remodeling.

For the first time, our data show that specific metabolic signatures are associated with heterogeneous cardiac remodeling trajectories after bariatric surgery. Although these analyses were exploratory and not designed to establish predictive biomarkers, our results represent a starting point for further studies to develop personalized care for obese patients undergoing bariatric surgery and metabolic unloading [44, 45].

In this context, the metabolomic changes identified in our cohort may indicate a shift toward a more balanced systemic and myocardial metabolic state. Evidence from human studies shows that bariatric surgery is linked to normalization of myocardial substrate metabolism, including decreased fatty acid uptake and better glucose utilization, along with concurrent improvements in cardiac structure and function [46]. The modulation of specific metabolite classes observed in our analysis aligns with alterations in pathways related to fatty acid metabolism, amino acid turnover, and mitochondrial function, all of which are essential for myocardial energetics and remodeling processes.

Because our analysis is based on circulating metabolites, it reflects systemic metabolic adaptations and does not allow direct inference on myocardial-specific metabolic processes. Therefore, any mechanistic interpretation linking metabolomic patterns to cardiac remodeling should be considered indirect and hypothesis-generating.

Importantly, the primary strength of this study lies not in the magnitude of average echocardiographic shifts but in the identification of coordinated metabolic signatures associated with distinct remodeling phenotypes. By integrating longitudinal metabolomics with structural trajectories, we provide evidence that systemic metabolic reprogramming parallels cardiac geometric adaptation in a biologically coherent manner. These observations should be considered hypothesis-generating and mechanistically informative rather than definitive evidence of population-wide structural normalization.

Conceptually, our results support a three-step biological sequence: (1) bariatric surgery is associated with abrupt systemic metabolic unloading and endocrine recalibration; (2) this triggers reprogramming of substrate handling pathways, including BCAA oxidation, fatty acid β-oxidation, ketone metabolism, and TCA cycle flux; and (3) in metabolically adaptable individuals, restoration of mitochondrial efficiency reduces hypertrophic energetic stress, allowing regression of concentric geometry. In contrast, individuals with persistent metabolic inflexibility exhibit attenuated structural recovery despite equivalent anthropometric changes. Framing cardiac remodeling within this metabolic adaptability paradigm provides a unifying explanation for the heterogeneity observed in our cohort.

### Limitations

Despite the novelty and translational relevance of our findings, several limitations must be acknowledged. First, this study was conducted at a single tertiary referral center and employed a prospective observational design, which inherently carries the risk of selection bias and limits the external generalizability of the results. Although we included a relatively large sample of severely obese individuals, the cohort was predominantly young and free from overt cardiovascular disease, our conclusions may not be directly applicable to older populations or to patients with advanced cardiometabolic comorbidities. On the other hand, the relatively modest sample size and magnitude of group-level echocardiographic changes limit the generalizability of our findings. Given the substantial interindividual variability, extrapolation to broader bariatric populations should be undertaken with caution. Larger multicenter cohorts with longer follow-up will be necessary to determine whether the metabolic–structural associations observed here translate into consistent population-level remodeling patterns. In addition, some subgroup analyses—particularly those based on remodeling trajectories—were conducted in relatively modest classes, which may increase statistical instability and limit the reproducibility of class-specific metabolomic signatures. Although repeated cross-validation, permutation testing, and confounder-adjusted analyses supported the robustness of the main multivariate patterns, these subgroup findings should be interpreted as exploratory and hypothesis-generating. Future studies in larger cohorts should further assess subgroup stability using resampling-based approaches such as bootstrapping. An additional limitation relates to potential confounding by pharmacological therapy. Although the prevalence of antihypertensive, statin, hypoglycemic, and other cardiovascular medications did not change significantly during follow-up, modifications in dosing or adherence could theoretically influence both myocardial structure and circulating metabolites. To avoid this, our analyses were specifically adjusted for medication, but we cannot completely disentangle drug-mediated effects from surgery-associated metabolic reprogramming. Future studies with randomized designs controlling for pharmacotherapy, will be necessary to isolate the independent contribution of metabolic remodeling to cardiac structural recovery. Second, the follow-up period was limited to 24 weeks, a timeframe that allowed us to capture early metabolic reprogramming and structural cardiac adaptations but did not permit assessment of long-term stability, reversibility, or potential relapse of remodeling. In addition, variability in data availability across time points resulted in differences in sample size between analyses. Although this reflects real-world clinical follow-up conditions, it may influence statistical power and generalizability. However, the consistency of multivariate results, validation metrics, and confounder-adjusted analyses supports the robustness of the main findings. Nevertheless, RWT improved significantly, changes in LVMI did not reach statistical significance at the whole-cohort level. This may reflect the relatively short follow-up duration, the young age of participants, and the modest baseline degree of remodeling. Therefore, conclusions regarding regression of myocardial mass should be interpreted cautiously. Our findings primarily support early geometric adaptation rather than definitive mass regression, and longer-term studies are needed to determine whether sustained metabolic reprogramming translates into statistically robust LVMI reduction. The use of distribution-based cut-offs for trajectory stratification, while biologically informative, may limit external reproducibility. Future studies employing predefined absolute thresholds or validated trajectory modeling approaches (e.g., latent class mixed modeling) would further strengthen phenotypic classification. Third, even though we applied rigorous untargeted metabolomic profiling and robust statistical modeling, the annotation of metabolites in untargeted GC–MS remains constrained by reference libraries and analytical standards availability, leading to incomplete or provisional identification of certain analytes. This may have prevented us from capturing the full spectrum of metabolic pathways relevant to cardiac remodeling. Additionally, metabolomics inherently reflects systemic, rather than organ-specific, biochemical states; therefore, the observed associations cannot support direct causality between metabolic shifts and myocardial recovery. Fourth, despite the use of multivariate analyses, including PLS-DA and LASSO regression, the possibility of overfitting cannot be fully excluded, especially given the relatively modest sample size compared with the large number of detected metabolites. Although we employed permutation testing, cross-validation, and synthetic data augmentation techniques to mitigate these concerns, the results should still be considered hypothesis-generating rather than definitive. Finally, while bariatric surgery offers a unique model of rapid metabolic and structural adaptation, the findings cannot necessarily be extrapolated to non-surgical weight-loss interventions or to populations with milder forms of obesity. Furthermore, emerging pharmacological strategies, such as GLP-1 receptor agonists and dual incretin therapies (e.g., tirzepatide), have demonstrated substantial efficacy in reducing body weight and improving cardiometabolic profiles and should be explored in future studies as either comparators to surgical interventions or as independent strategies for assessing cardiac remodeling. Taken together, these limitations highlight the need for multicenter, prospective, and mechanistically focused studies integrating metabolomics with advanced imaging and molecular analyses consistent with, refined, and extend to our observations. Such studies will be essential to validate the predictive utility of pre-intervention metabolic fingerprints, to disentangle weight-loss-dependent and weight-loss-independent effects, and ultimately to advance the development of precision cardiometabolic strategies for patients with obesity-related cardiac remodeling.

## Conclusion

In this longitudinal cohort of individuals undergoing bariatric surgery, early geometric cardiac adaptation—primarily reflected by reductions in RWT and heterogeneous directional changes in LVMI—was associated with coordinated systemic metabolic reprogramming. At the cohort level, changes in LVMI were modest and did not reach statistical significance, underscoring the importance of distinguishing geometric from mass-related remodeling. The observed metabolomic signatures reflect systemic metabolic changes and cannot be used to infer myocardial-specific mechanisms. Although distinct remodeling trajectories were associated with coherent metabolic patterns, these findings should be interpreted as exploratory and hypothesis-generating. Overall, this study highlights a potential link between systemic metabolic adaptation and early cardiac geometric changes after bariatric surgery, warranting further investigation in larger, mechanistically focused studies.

## Supplementary Information

Supplementary material 1.

## Data Availability

The datasets analyzed during the current study are available from the corresponding author on reasonable request.

## Abbreviations

ALT: Alanine aminotransferase
AST: Aspartate aminotransferase
BMI: Body mass index
BNP: B-type natriuretic peptide
BSA: Body surface area
BCAA: Branched-chain amino acid
CBC: Complete blood count
CRP: C-reactive protein
ECG: Electrocardiogram
GC–MS: Gas chromatography–mass spectrometry
HbA1c: Glycated hemoglobin
HDL: High-density lipoprotein
HFpEF: Heart failure with preserved ejection fraction
IVSd: Interventricular septum at end-diastole
LDL: Low-density lipoprotein
LASSO: Least absolute shrinkage and selection operator
LAVi: Left atrial volume index
LV: Left ventricle
LVEDV: Left ventricular end-diastolic volume
LVESV: Left ventricular end-systolic volume
LVH: Left ventricular hypertrophy
LVCR: Left ventricular concentric remodeling
LVMI: Left ventricular mass index
MSI: Metabolomics standards initiative
NT-proBNP: N-terminal pro-B-type natriuretic peptide
PAPs: Pulmonary artery systolic pressure
PCA: Principal component analysis
PLS-DA: Partial least squares discriminant analysis
RWT: Relative wall thickness
SCFA: Short-chain fatty acid
SG: Sleeve gastrectomy
TAPSE: Tricuspid annular plane systolic excursion
TCA: Tricarboxylic acid
TMAO: Trimethylamine-N-oxide
