# Supplementary material for: Cardiac remodeling after bariatric surgery associates with systemic metabolic reprogramming: a longitudinal untargeted metabolomics study
Source: Cardiovasc Diabetol. 2026 Jun 2;25:246. doi: 10.1186/s12933-026-03231-y (PMC13491655; doi:10.1186/s12933-026-03231-y)
Supplement: Supplementary file 1 [file 12933_2026_3231_MOESM1_ESM.docx]

**Supplementary Material**

**Supplementary Materials Overview**

To enhance transparency and reproducibility, detailed methodological procedures and extended results are provided in the Supplementary Materials. A schematic overview of the study design and analytical workflow is presented in Supplementary Figure S0, illustrating the longitudinal structure of the cohort, data acquisition processes, and the statistical modeling strategy adopted.

Subsequent sections provide in-depth descriptions of data preprocessing, normalization, confounder adjustment, and residualization procedures (Supplementary Methods S1), followed by additional analyses supporting the robustness of the results, including residualization assessments and full metabolite-level statistical outputs with multiple-testing correction (Supplementary Tables S1–S7).

**Supplementary Figure S0. Schematic overview of the study design and analytical workflow.** This figure summarizes the longitudinal study design, data acquisition, and analytical pipeline used in the present study. A cohort of 127 patients undergoing bariatric surgery was followed at baseline (T0), 4 weeks (T4), 12 weeks (T12), and 24 weeks (T24). Serum metabolomics (GC–MS) and echocardiographic measurements (LVMI, RWT) were collected at each timepoint. Metabolomics data were preprocessed (peak alignment, normalization, log transformation, autoscaling, and feature filtering) and analyzed using multivariate statistical approaches (PLS-DA, cross-validation, permutation testing, VIP-based selection, and LASSO regression), with false discovery rate (FDR) correction applied. Patients were stratified into cardiac remodeling phenotypes (Class 0: stable geometry; Class 1: early reverse remodeling; Class 2: persistent remodeling), and associated metabolic signatures and baseline predictors were identified.

**S1. Metabolomic Data Preprocessing**

Raw GC–MS chromatograms were aligned using the parametric time warping (PTW) algorithm to correct for retention time shifts across samples. Peak detection, deconvolution, and integration were performed using standardized pipelines to ensure consistency.

Metabolite intensities were normalized to the internal standard (2-isopropyl malic acid) to account for analytical variability. Data were subsequently subjected to generalized logarithmic transformation (glog), defined as:

$$glog(x)=\log\left( \frac{x+\sqrt{x^{2}+\lambda^{2}}}{2} \right)$$

where $\lambda$is a regularization parameter that stabilizes variance across low-intensity features.

Autoscaling was then applied:

$$x^{'}=\frac{x-\mu}{\sigma}$$

where $\mu$and $\sigma$represent the mean and standard deviation of each metabolite across samples, ensuring equal weighting in multivariate analyses.

Metabolites detected in fewer than 80% of samples were excluded to reduce sparsity and improve statistical robustness.

**S2. Supervised Multivariate Modeling and Validation Strategy**

Supervised classification analyses were performed using Partial Least Squares–Discriminant Analysis (PLS-DA), a latent variable method that models the relationship between a predictor matrix $\mathbf{X}\in\mathbb{R}^{n\times p}$(metabolites) and a categorical response variable $\mathbf{Y}\in\mathbb{R}^{n}$(class labels).

PLS-DA decomposes $\mathbf{X}$into latent structures:

$$\mathbf{X}=\mathbf{T}\mathbf{P}^{\top}+\mathbf{E}$$

where:

- $\mathbf{T}$= score matrix (latent components),
- $\mathbf{P}$= loading matrix,
- $\mathbf{E}$= residual matrix.

The latent components are selected to maximize covariance between $\mathbf{T}$and the response variable $\mathbf{Y}$.

**Cross-validation**

Model performance and tuning were evaluated using **7-fold cross-validation with repeated resampling** implemented via the caret framework in R. In each iteration:

- the dataset was partitioned into 7 subsets,
- 6 subsets were used for training,
- 1 subset was used for validation,
- the process was repeated across folds and multiple repetitions to reduce variance in performance estimates.

Classification performance was summarized using cross-validated accuracy and balanced accuracy where appropriate.

**Permutation testing**

To assess the statistical significance of class separation and evaluate the risk of overfitting, permutation testing was performed using **1000 random permutations** of class labels.

For each permutation $i$, class labels were randomly reassigned:

$$\mathbf{Y}^{\left( i \right)}=\pi_{i}(\mathbf{Y})$$

and the model was retrained to obtain a null distribution of performance metrics. The empirical p-value was computed as:

$$p=\frac{1+\sum_{i=1}^{N} \mathbb{I}(M_{i}\geq M_{obs})}{N+1}$$

where:

- $M_{obs}$= observed model performance,
- $M_{i}$= performance under permutation,
- $N$= number of permutations.

**Class imbalance handling**

In the presence of class imbalance, **SMOTE (Synthetic Minority Over-sampling Technique)** was applied within the training folds only. This ensures that synthetic samples are generated exclusively from training data, preventing information leakage into validation sets.

**Interpretation framework**

PLS-DA models were interpreted as **exploratory multivariate tools for identifying structured variation and class-associated patterns**, rather than fully validated predictive classifiers.

**S3. Feature Selection and Variable Importance**

Variable Importance in Projection (VIP) scores were computed to quantify the contribution of each metabolite to class discrimination. VIP scores are defined as:

$$VIP_{j}=\sqrt{p\cdot\frac{\sum_{a=1}^{A} SSY_{a}\cdot w_{ja}^{2}}{\sum_{a=1}^{A} SSY_{a}}}$$

where:

- $p$= number of variables,
- $SSY_{a}$= explained variance in $Y$by component $a$,
- $w_{ja}$= weight of variable $j$in component $a$.

Metabolites with $VIP>1.5$were considered relevant contributors to class separation.

Given the high dimensionality of metabolomic data, VIP-based selection was used as a **descriptive prioritization strategy**, not as an independent validation of biomarker stability.

**S4. LASSO Regression Modeling**

To identify metabolite features associated with continuous echocardiographic parameters, Least Absolute Shrinkage and Selection Operator (LASSO) regression was applied.

Given a predictor matrix $\mathbf{X}$and response vector $\mathbf{y}$, LASSO solves:

$$\hat{\boldsymbol{\beta}}=\arg\min_{\boldsymbol{\beta}}\left( \frac{1}{2n}\parallel\mathbf{y}-\mathbf{X}\boldsymbol{\beta}\parallel_{2}^{2}+\alpha\parallel\boldsymbol{\beta}\parallel_{1} \right)$$

where:

- $\parallel\boldsymbol{\beta}\parallel_{1}=\sum\mid\beta_{j}\mid$enforces sparsity,
- $\alpha$controls the degree of regularization.

**Model training and validation**

- Data were scaled using MinMax normalization.
- The dataset was split into **training (70%) and testing (30%) subsets**.
- The regularization parameter $\alpha$was optimized via cross-validation within the training set.
- Model performance was evaluated on the independent test set using regression metrics (e.g., $R^{2}$, mean squared error).

Metabolites with non-zero coefficients in the optimized model were retained as candidate predictors.

Given the dimensionality of the dataset relative to sample size, LASSO outputs were interpreted as **feature selection results** rather than validated predictive models.

**S5. Residualization for Confounder Adjustment**

To evaluate the potential influence of clinical confounders on metabolomic profiles, a residualization approach was applied.

For each metabolite $y_{j}$, a linear regression model was fitted:

$$y_{j}=\beta_{0}+\sum_{k=1}^{K} \beta_{k}C_{k}+\epsilon$$

where:

- $C_{k}$represents confounding variables,
- $\epsilon$represents the residual component.

Residuals were computed as:

$$\hat{\epsilon}=y_{j}-\hat{y}_{j}$$

and used as adjusted metabolite values for downstream analyses.

**Confounders included**

The following variables were included based on clinical relevance:

- family history of cardiovascular disease
- hypertension status
- statin therapy
- antihypertensive therapy

Categorical variables were encoded using one-hot encoding.

**Missing data handling**

Missing values in confounders were handled as follows:

- continuous variables: median imputation
- categorical variables: mode imputation

**Implementation details**

Residualization was performed independently for each metabolite using linear regression models. The adjusted dataset was then used to repeat supervised multivariate analyses (PLS-DA), enabling assessment of whether metabolomic signatures persisted independently of confounding factors.

**S6. Impact of Confounder Adjustment on Multivariate Models**

To evaluate the robustness of metabolomic signatures with respect to potential clinical confounders, all supervised multivariate analyses were repeated using residualized data as described in Section S5, and directly compared with models built on the original (non-residualized) dataset.

Across all time points (T0, T4, and T12), PLS-DA score plots showed highly consistent group separation patterns between residualized and non-residualized data, with no evident changes in clustering structure or class discrimination.

Model performance metrics, including cross-validated accuracy, R², and Q², exhibited comparable trends across increasing numbers of components in both analytical settings. Minor variations were observed but remained within a narrow range, indicating negligible impact of confounder adjustment on predictive performance.

Permutation testing further confirmed that classification performance remained significantly higher than expected under random class assignment in both residualized and non-residualized models, with comparable empirical p-values across conditions.

In addition, metabolite ranking based on VIP scores and fold-change analysis showed strong concordance between residualized and original datasets, and pathway enrichment analysis yielded highly overlapping biological interpretations.

Overall, these results demonstrate that the metabolomic signatures associated with cardiac remodeling are robust and not substantially influenced by the considered clinical confounders, supporting their biological relevance rather than reflecting confounding-driven effects.

**Figure S1. Multivariate analysis based on residualized metabolomic data for time-point comparisons** (corresponding to Figure 3 in the main text). Panels (A1–A3) show PLS-DA score plots at T0, T4, and T12. Panels (B1–B3) report cross-validated model performance (accuracy, R², Q²), and panels (C1–C3) show permutation test results. Results are highly consistent with those obtained from non-residualized data, indicating that group separation and model performance are not driven by the considered confounders.

**Figure S1. Class-specific multivariate analysis based on residualized metabolomic data** (corresponding to Figure 4 in the main text). Panels (A1–C1) show PLS-DA score plots for Class 0, Class 1, and Class 2, respectively. Panels (A2–C2) display metabolite fold-change profiles, while panels (A3–C3) show permutation test results and model validation metrics. Comparable classification performance and metabolite patterns between residualized and non-residualized data confirm the robustness of class-specific metabolic signatures.

**Figure S2. Baseline predictive modeling and pathway analysis based on residualized metabolomic data (corresponding to Figure 5 in the main text).** Panel (A) shows PLS-DA score plot, panel (B) reports model performance metrics, panel (C) shows permutation test results, panel (D) displays differential metabolite profiles, and panel (E) presents pathway enrichment analysis. The consistency of results with those obtained from non-residualized data supports the stability of baseline metabolic signatures after adjustment for confounders.

**Supplementary Figure S3. Validation and performance assessment of the PLS-DA models.** (A) Three-dimensional score plot of the PLS-DA model based on the first three latent variables (LV1–LV3), illustrating the separation between groups. Each point represents an individual sample, color-coded according to the direction of change (Dec: decrease; Inc: increase). The shaded region highlights the main cluster distribution.
(B) Model performance metrics across an increasing number of latent variables, including accuracy, explained variance (R²), and predictive ability (Q²). The progressive improvement of model performance with additional components supports the robustness of the multivariate classification. (C) Permutation test (1000 permutations) evaluating the statistical significance of the PLS-DA model. The observed model statistic is indicated by the red arrow and falls outside the null distribution, confirming that the model performance is not due to random chance.

**S7. Comprehensive metabolite-level statistical analysis**

A comprehensive list of metabolite-level statistical results, including raw p-values and false discovery rate (FDR)-adjusted q-values, is provided in **Table S1–S5**.

At baseline (T0), although several metabolites showed nominal statistical significance (p < 0.05), none remained significant after multiple testing correction (FDR > 0.10), indicating the absence of strong pre-existing metabolomic separation between groups.

At 4 weeks post-intervention (T4), multiple metabolites reached statistical significance after FDR correction (q < 0.05), including intermediates involved in carbohydrate metabolism (e.g., deoxyribose), amino acid metabolism (e.g., 2-aminobutyric acid), and organic acid pathways (e.g., succinic acid and glycolic acid), suggesting the emergence of structured metabolic divergence associated with early cardiac remodeling.

At 12 weeks (T12), a larger number of metabolites remained significant after multiple testing correction (q < 0.01), including short-chain fatty acids (valeric and butyric acid), branched-chain amino acid derivatives (3-methyl-2-oxovaleric acid, ketoleucine), and tricarboxylic acid (TCA) cycle intermediates (malic acid). These results indicate a progressive consolidation of metabolomic differences over time.

Class-based analyses further highlighted heterogeneity in metabolic responses. In Class 0, several metabolites remained significant after FDR correction (q < 0.05), including lactic acid and N-formylglycine, suggesting robust metabolic adaptation primarily associated with weight loss. In contrast, Class 1 did not show significant metabolites after multiple testing correction, consistent with greater variability or smaller effect sizes. Class 2 showed limited but detectable signals (q < 0.10), suggesting partial metabolic divergence.

Baseline predictive analyses (Figure 5) identified several metabolites with nominal significance (p < 0.01), but none survived stringent multiple testing correction (q ≥ 0.05), indicating that baseline signatures should be interpreted as candidate predictors rather than definitive biomarkers.

Overall, these results demonstrate that metabolomic separation becomes progressively stronger after intervention and is most robust at later time points, while baseline differences remain modest after correction for multiple comparisons.

**Table S1. Metabolite-level statistical results at baseline (T0) for Figure 3.** Metabolites are reported with corresponding raw p-values and false discovery rate (FDR)-adjusted q-values. Although several metabolites showed nominal significance (p < 0.05), none remained significant after multiple testing correction (q ≥ 0.10), indicating the absence of statistically robust metabolomic differences at baseline.

| Metabolites | Raw p-value | FDR-adjusted q-value | Significance level |
| --- | --- | --- | --- |
| Sugar_17 | 0.0051381 | 0.24404 |  |
| Glyceric acid | 0.0055279 | 0.24404 |  |
| Sugar_1 | 0.0061105 | 0.24404 |  |
| Sugar_9 | 0.0087329 | 0.24404 |  |
| L-Threonic acid | 0.0097245 | 0.24404 |  |
| 2-Hydroxy-2-methylbutyric acid | 0.010473 | 0.24404 |  |
| Ribose II | 0.011296 | 0.24404 |  |
| Norvaline | 0.011484 | 0.24404 |  |
| Pentanedioic acid | 0.013822 | 0.26108 |  |
| meso-Erythritol | 0.028862 | 0.49066 |  |
| Serine | 0.032937 | 0.49665 |  |
| Diethylene glycol | 0.036523 | 0.49665 |  |
| Leucine | 0.037979 | 0.49665 |  |
| Allose | 0.047281 | 0.50505 |  |
| Sugar_13 | 0.047474 | 0.50505 |  |

**Table S2. Metabolite-level statistical results at 4 weeks post-intervention (T4) for Figure 3.** Metabolites are reported with corresponding raw p-values and FDR-adjusted q-values. Several metabolites remained statistically significant after multiple testing correction (q < 0.05), indicating the emergence of structured metabolomic differences associated with early cardiac remodeling. Significance levels are based on FDR-adjusted q-values: * q < 0.10, ** q < 0.05, *** q < 0.01.

| Metabolites | Raw p-value | FDR-adjusted q-value | Significance level |
| --- | --- | --- | --- |
| Unknown_2 | 0.00016728 | 0.010754 | ** |
| Deoxyribose | 0.00029737 | 0.010754 | ** |
| Unknown_1 | 0.00040967 | 0.010754 | ** |
| Hydroxybutyric acid | 0.00043702 | 0.010754 | ** |
| 2-Butenedioic acid | 0.00047414 | 0.010754 | ** |
| Edetic Acid; 4TMS derivative | 0.00049326 | 0.010754 | ** |
| Urea | 0.00050108 | 0.010754 | ** |
| 2-Methylbenzoic acid | 0.00056424 | 0.010754 | ** |
| Sugar_7 | 0.00056931 | 0.010754 | ** |
| Succinic acid | 0.00063662 | 0.010823 | ** |
| Mannose II | 0.00081189 | 0.012547 | ** |
| Unknown_16 | 0.0009948 | 0.014093 | ** |
| 2-Aminobutyric acid | 0.0014847 | 0.019416 | ** |
| Hydroxyisobutyric acid | 0.0018397 | 0.020305 | ** |
| Glycolic acid | 0.0018851 | 0.020305 | ** |

**Table S3. Metabolite-level statistical results at 12 weeks post-intervention (T12) for Figure 3.** Metabolites are reported with corresponding raw p-values and FDR-adjusted q-values. A substantial number of metabolites remained significant after multiple testing correction (q < 0.01), reflecting a consolidation of metabolomic divergence over time. Significance levels are based on FDR-adjusted q-values: * q < 0.10, ** q < 0.05, *** q < 0.01.

| Metabolites | Raw p-value | FDR-adjusted q-value | Significance level |
| --- | --- | --- | --- |
| Valeric acid | 1.66E-07 | 1.79E-05 | *** |
| 3-Methyl-2-oxovaleric acid | 2.37E-07 | 1.79E-05 | *** |
| Ketoleucine | 3.88E-07 | 1.79E-05 | *** |
| Glycolic acid | 4.21E-07 | 1.79E-05 | *** |
| Benzoic Acid | 6.34E-07 | 2.16E-05 | *** |
| Malic acid | 1.02E-06 | 2.55E-05 | *** |
| N-Formylglycine | 1.11E-06 | 2.55E-05 | *** |
| 2-Hydroxycaproic acid | 1.24E-06 | 2.55E-05 | *** |
| Gluconic acid | 1.45E-06 | 2.55E-05 | *** |
| Sarcosine | 1.50E-06 | 2.55E-05 | *** |
| Unknown_19 | 2.07E-06 | 3.20E-05 | *** |
| Alanine | 2.58E-06 | 3.59E-05 | *** |
| Butyric acid | 2.74E-06 | 3.59E-05 | *** |
| Sebacic acid | 3.25E-06 | 3.66E-05 | *** |
| 2-Aminobutyric acid | 3.99E-06 | 3.66E-05 | *** |

**Table S4. Metabolite-level statistical results for Class 0 (stable geometry) in Figure 4.** Metabolites are reported with corresponding raw p-values and FDR-adjusted q-values. Several metabolites remained significant after multiple testing correction (q < 0.05), indicating robust metabolic changes primarily associated with weight loss in individuals with stable cardiac geometry. Significance levels are based on FDR-adjusted q-values: * q < 0.10, ** q < 0.05, *** q < 0.01.

| Metabolites | Raw p-value | FDR-adjusted q-value | Significance level |
| --- | --- | --- | --- |
| Lactic Acid | 2.46E-09 | 4.11E-07 | *** |
| N-Formylglycine | 0.00053505 | 0.044676 | ** |
| Arabinose III | 0.0014522 | 0.066871 | * |
| Tymidine | 0.0016017 | 0.066871 | * |
| Leucine | 0.0021241 | 0.070945 | * |
| Threonine | 0.0034585 | 0.088528 | * |
| Valine | 0.004154 | 0.088528 | * |
| Fatty acid_6 | 0.0042409 | 0.088528 | * |
| Fatty acid_5 | 0.0057219 | 0.10617 |  |
| Fatty acid_7 | 0.00693 | 0.11573 |  |
| Fatty acid_8 | 0.0077509 | 0.11767 |  |
| Deoxyglucose | 0.010179 | 0.13425 |  |
| Nonanoic acid | 0.011218 | 0.13425 |  |
| Urea | 0.011645 | 0.13425 |  |
| Arabinose IV | 0.012699 | 0.13425 |  |

**Table S5. Metabolite-level statistical results for Class 1 (early remodelers) in Figure 4.** Metabolites are reported with corresponding raw p-values and FDR-adjusted q-values. Although several metabolites showed nominal significance (p < 0.05), none remained significant after multiple testing correction, indicating higher variability and/or smaller effect sizes in this subgroup. Significance levels are based on FDR-adjusted q-values: * q < 0.10, ** q < 0.05, *** q < 0.01.

| Metabolites | Raw p-value | FDR-adjusted q-value | Significance level |
| --- | --- | --- | --- |
| Lactic Acid | 0.0016369 | 0.14513 |  |
| 2;3-Butanediol | 0.0017381 | 0.14513 |  |
| 2-Furanacetic acid | 0.0030772 | 0.1713 |  |
| Hydroxyisobutyric acid | 0.0083065 | 0.24283 |  |
| Unknown_11 | 0.012524 | 0.24283 |  |
| Unknown_12 | 0.013869 | 0.24283 |  |
| Unknown_13 | 0.014862 | 0.24283 |  |
| Succinic acid | 0.014964 | 0.24283 |  |
| Sarcosine | 0.016806 | 0.24283 |  |
| Leucine | 0.017337 | 0.24283 |  |
| 2-Butenedioic acid | 0.01749 | 0.24283 |  |
| Unknown_2 | 0.021578 | 0.24283 |  |
| Unknown_1 | 0.021881 | 0.24283 |  |
| Methylmalonic acid | 0.024885 | 0.24283 |  |
| Methylsuccinic acid | 0.025358 | 0.24283 |  |

**Table S6. Metabolite-level statistical results for Class 2 (non-remodelers) in Figure 4.** Metabolites are reported with corresponding raw p-values and FDR-adjusted q-values. A limited number of metabolites showed significance after multiple testing correction at a relaxed threshold (q < 0.10), suggesting partial metabolic divergence in individuals with delayed or absent cardiac remodeling. Significance levels are based on FDR-adjusted q-values: * q < 0.10, ** q < 0.05, *** q < 0.01.

| Metabolites | Raw p-value | FDR-adjusted q-value | Significance level |
| --- | --- | --- | --- |
| Sugar_7 | 0.00012078 | 0.02017 | ** |
| Pyrogallol | 0.0015028 | 0.093223 | * |
| Fatty acid_2 | 0.0024274 | 0.093223 | * |
| Unknown_2 | 0.0027165 | 0.093223 | * |
| Glycerol | 0.0027911 | 0.093223 | * |
| Sugar_12 | 0.0037393 | 0.10021 |  |
| Unknown_5 | 0.0047283 | 0.10021 |  |
| Unknown_20 | 0.0058823 | 0.10021 |  |
| Unknown_17 | 0.0064525 | 0.10021 |  |
| 6-Hydroxy-.alpha.-methylnaphthaleneacetic acid | 0.0064605 | 0.10021 |  |
| Pyroglutamic acid | 0.0066004 | 0.10021 |  |
| Unknown_23 | 0.0073226 | 0.10191 |  |
| Arabinose III | 0.0093624 | 0.10311 |  |
| Unknown_10 | 0.0095581 | 0.10311 |  |
| Unknown_4 | 0.0097173 | 0.10311 |  |

**Table S7. Baseline metabolite-level statistical results associated with subsequent cardiac remodeling trajectories (Figure 5).** Metabolites are reported with corresponding raw p-values and FDR-adjusted q-values. Several metabolites showed nominal statistical significance; however, none remained significant after stringent multiple testing correction (q ≥ 0.05), indicating that baseline metabolic differences should be interpreted as candidate signals rather than validated predictors. Significance levels are based on FDR-adjusted q-values: * q < 0.10, ** q < 0.05, *** q < 0.01.

| Metabolites | Raw p-value | FDR-adjusted q-value | Significance level |
| --- | --- | --- | --- |
| Sugar_13 | 0.0005615 | 0.083219 | * |
| Sugar_22 | 0.0024268 | 0.083219 | * |
| Mannobiose | 0.0025714 | 0.083219 | * |
| Rhamnose I | 0.0028202 | 0.083219 | * |
| Thymol | 0.0041393 | 0.083219 | * |
| Sugar_4 | 0.0042807 | 0.083219 | * |
| Sugar_18 | 0.0042969 | 0.083219 | * |
| Sugar_17 | 0.0048537 | 0.083219 | * |
| Sugar_15 | 0.004969 | 0.083219 | * |
| Arabinose II | 0.0049832 | 0.083219 | * |
| Asparagine | 0.0063986 | 0.089666 | * |
| Deoxyglucose | 0.0064431 | 0.089666 | * |
| Fatty acid_5 | 0.0072863 | 0.093601 | * |
| Sugar_2 | 0.0091295 | 0.10237 |  |
| Fatty acid_8 | 0.0096131 | 0.10237 |  |

**S8. Pathway Enrichment Analysis**

Metabolic pathway enrichment analysis was conducted using Metabolomics Pathway Analysis (MetPa), based on KEGG pathway annotations.

Enrichment was evaluated using a combination of:

- over-representation analysis (ORA)
- pathway topology analysis

Pathways were ranked based on enrichment scores and statistical significance, allowing identification of biologically relevant metabolic processes associated with cardiac remodeling phenotypes.

**Supplementary Table S8. Comprehensive list of detected metabolites included in the analysis.**
The table reports all metabolite features retained after preprocessing (presence ≥80% across samples), including fully identified compounds (MSI level 1), class-level annotations (MSI level 3), and unknown features (MSI level 4). For each metabolite, median values and interquartile ranges (IQR) are provided to describe distribution within the study cohort. This table ensures full transparency of the metabolomic dataset used for downstream statistical and multivariate analyses.

| Metabolite_Name | MSI_Level | Annotation_Type | Median | Interquartile Range (IQR) |
| --- | --- | --- | --- | --- |
| 1,5-Anhydrohexitol | MSI 1 | Identified metabolite | 0.105931 | 0.101598–0.20656 |
| 2,3-Butanediol | MSI 1 | Identified metabolite | 0.012393 | 0.010509–0.046283 |
| 2-Aminobutyric acid | MSI 1 | Identified metabolite | 0.014606 | 0.01119–0.052937 |
| 2-Butenedioic acid | MSI 1 | Identified metabolite | 0.013449 | 0.010939–0.052892 |
| 2-Furanacetic acid | MSI 1 | Identified metabolite | 0.020153 | 0.013781–0.064706 |
| 2-Hydroxy-2-methylbutyric acid | MSI 1 | Identified metabolite | 0.023518 | 0.019625–0.0753 |
| 2-Hydroxycaproic acid | MSI 1 | Identified metabolite | 0.009374 | 0.00817–0.03628 |
| 2-Hydroxyglutaric acid | MSI 1 | Identified metabolite | 0.015051 | 0.012195–0.050162 |
| 2-Hydroxyisocaproic acid | MSI 1 | Identified metabolite | 0.007107 | 0.00575–0.029448 |
| 2-Methylbenzoic acid | MSI 1 | Identified metabolite | 0.006303 | 0.005251–0.025228 |
| 3-Acetoxybutyric acid | MSI 1 | Identified metabolite | 0.020767 | 0.017302–0.067376 |
| 3-Methyl-2-oxovaleric acid | MSI 1 | Identified metabolite | 0.018358 | 0.014448–0.062586 |
| 3-Phenyllactic acid | MSI 1 | Identified metabolite | 0.004578 | 0.003703–0.017015 |
| 5-Oxoproline | MSI 1 | Identified metabolite | 0.042652 | 0.029011–0.060177 |
| 5-Phenylvaleric acid | MSI 1 | Identified metabolite | 0.030443 | 0.026602–0.12353 |
| 6-Hydroxy-.alpha.-methylnaphthaleneacetic acid | MSI 1 | Identified metabolite | 0.058236 | 0.048493–0.177928 |
| Acetamide | MSI 1 | Identified metabolite | 0.012455 | 0.009149–0.038007 |
| Acetoacetic acid | MSI 1 | Identified metabolite | 0.00505 | 0.003775–0.020003 |
| Alanine | MSI 1 | Identified metabolite | 0.020999 | 0.019017–0.071607 |
| Allofuranose | MSI 1 | Identified metabolite | 0.022686 | 0.016885–0.083082 |
| Allose | MSI 1 | Identified metabolite | 0.003098 | 0.002629–0.012977 |
| Aminomalonic acid | MSI 1 | Identified metabolite | 0.016766 | 0.013261–0.047512 |
| Anisuric acid | MSI 1 | Identified metabolite | 0.008354 | 0.007814–0.029687 |
| Arabinitol | MSI 1 | Identified metabolite | 0.015279 | 0.013063–0.053828 |
| Arabinose I | MSI 1 | Identified metabolite | 0.024106 | 0.019695–0.091134 |
| Arabinose II | MSI 1 | Identified metabolite | 0.024055 | 0.017094–0.072709 |
| Arabinose III | MSI 1 | Identified metabolite | 0.009112 | 0.007316–0.033233 |
| Arabinose IV | MSI 1 | Identified metabolite | 0.006423 | 0.0046–0.02274 |
| Asparagine | MSI 1 | Identified metabolite | 0.049988 | 0.03674–0.15255 |
| Aspartic acid | MSI 1 | Identified metabolite | 0.008184 | 0.007071–0.031504 |
| Benzoic Acid | MSI 1 | Identified metabolite | 0.018816 | 0.015558–0.076942 |
| Butanedioic acid | MSI 1 | Identified metabolite | 0.015255 | 0.01287–0.044831 |
| Butyric acid | MSI 1 | Identified metabolite | 0.005308 | 0.004454–0.020669 |
| Deoxyglucose | MSI 1 | Identified metabolite | 0.015245 | 0.011927–0.072973 |
| Deoxyribose | MSI 1 | Identified metabolite | 0.005328 | 0.004342–0.019846 |
| Diethylene glycol | MSI 1 | Identified metabolite | 0.196976 | 0.047854–0.444 |
| Edetic Acid | MSI 1 | Identified metabolite | 0.726215 | 0.314827–1.874645 |
| Erythrose I | MSI 1 | Identified metabolite | 0.003646 | 0.003128–0.014985 |
| Erythrose II | MSI 1 | Identified metabolite | 0.009232 | 0.007857–0.036702 |
| Fatty acid_1 | MSI 3 | Class-level annotation | 0.040764 | 0.035065–0.110812 |
| Fatty acid_2 | MSI 3 | Class-level annotation | 0.067455 | 0.029088–0.133675 |
| Fatty acid_3 | MSI 3 | Class-level annotation | 0.227564 | 0.15298–0.298047 |
| Fatty acid_4 | MSI 3 | Class-level annotation | 0.230727 | 0.164821–0.303708 |
| Fatty acid_5 | MSI 3 | Class-level annotation | 0.020133 | 0.015522–0.065406 |
| Fatty acid_6 | MSI 3 | Class-level annotation | 0.025781 | 0.020207–0.081961 |
| Fatty acid_7 | MSI 3 | Class-level annotation | 0.016673 | 0.012908–0.053626 |
| Fatty acid_8 | MSI 3 | Class-level annotation | 0.010986 | 0.008738–0.03579 |
| Fatty acid_9 | MSI 3 | Class-level annotation | 0.070533 | 0.038177–0.096882 |
| Galacturonic acid | MSI 1 | Identified metabolite | 0.006674 | 0.00533–0.025991 |
| Gallic acid | MSI 1 | Identified metabolite | 0.019852 | 0.016737–0.067975 |
| Gluconic acid | MSI 1 | Identified metabolite | 0.028412 | 0.024886–0.105858 |
| Glucose | MSI 1 | Identified metabolite | 1.987079 | 1.686148–2.345914 |
| Glyceric acid | MSI 1 | Identified metabolite | 0.018147 | 0.015021–0.061756 |
| Glycerol | MSI 1 | Identified metabolite | 0.02635 | 0.02392–0.065888 |
| Glycine | MSI 1 | Identified metabolite | 0.031417 | 0.021721–0.101532 |
| Glycolic acid | MSI 1 | Identified metabolite | 0.032122 | 0.024645–0.116247 |
| Heptanoic acid | MSI 1 | Identified metabolite | 0.049376 | 0.040828–0.07945 |
| Hexanedioic acid | MSI 1 | Identified metabolite | 0.006523 | 0.004961–0.022973 |
| Hydroxybutyric acid | MSI 1 | Identified metabolite | 0.012579 | 0.010409–0.050197 |
| Hydroxyisobutyric acid | MSI 1 | Identified metabolite | 0.019782 | 0.018195–0.046845 |
| Hydroxylamine | MSI 1 | Identified metabolite | 0.015266 | 0.012157–0.057332 |
| Isoleucine | MSI 1 | Identified metabolite | 0.033865 | 0.031759–0.096781 |
| Ketoleucine | MSI 1 | Identified metabolite | 0.013249 | 0.011415–0.05005 |
| L-Threonic acid | MSI 1 | Identified metabolite | 0.020272 | 0.016884–0.070745 |
| Lactic Acid | MSI 1 | Identified metabolite | 0.382524 | 0.267893–1.079262 |
| Lactulose | MSI 1 | Identified metabolite | 0.004986 | 0.004296–0.020679 |
| Leucine | MSI 1 | Identified metabolite | 0.066317 | 0.045107–0.181704 |
| Malic acid | MSI 1 | Identified metabolite | 0.005455 | 0.004899–0.020256 |
| Maltose | MSI 1 | Identified metabolite | 0.009327 | 0.007633–0.033768 |
| Mannitol I | MSI 1 | Identified metabolite | 0.018112 | 0.013947–0.066618 |
| Mannitol II | MSI 1 | Identified metabolite | 0.007426 | 0.005933–0.02855 |
| Mannitol III | MSI 1 | Identified metabolite | 0.005413 | 0.004524–0.019842 |
| Mannobiose | MSI 1 | Identified metabolite | 0.024736 | 0.017599–0.074059 |
| Mannose I | MSI 1 | Identified metabolite | 0.030459 | 0.024178–0.095338 |
| Mannose II | MSI 1 | Identified metabolite | 0.003792 | 0.003308–0.015116 |
| meso-Erythritol | MSI 1 | Identified metabolite | 0.020318 | 0.015582–0.062772 |
| Methionine | MSI 1 | Identified metabolite | 0.011158 | 0.008702–0.034123 |
| Methylmalonic acid | MSI 1 | Identified metabolite | 0.022941 | 0.018197–0.079084 |
| Methylsuccinic acid | MSI 1 | Identified metabolite | 0.007937 | 0.00642–0.029204 |
| Myo-Inositol | MSI 1 | Identified metabolite | 0.035409 | 0.030163–0.08729 |
| Myristic acid | MSI 1 | Identified metabolite | 0.020475 | 0.016812–0.074851 |
| N-acetyll-D-glucosamine | MSI 1 | Identified metabolite | 0.00984 | 0.008559–0.041804 |
| N-Formylglycine | MSI 1 | Identified metabolite | 0.02296 | 0.019422–0.077178 |
| Nonanoic acid | MSI 1 | Identified metabolite | 0.026909 | 0.022585–0.088153 |
| Norvaline | MSI 1 | Identified metabolite | 0.036517 | 0.029328–0.085197 |
| o-Toluic acid | MSI 1 | Identified metabolite | 0.01359 | 0.011162–0.053338 |
| Octadecanoic acid | MSI 1 | Identified metabolite | 0.003327 | 0.002839–0.013771 |
| Palmitic Acid | MSI 1 | Identified metabolite | 0.098288 | 0.067894–0.160424 |
| Pantothenic acid | MSI 1 | Identified metabolite | 0.021021 | 0.016671–0.079918 |
| Pentanedioic acid | MSI 1 | Identified metabolite | 0.089697 | 0.058208–0.150372 |
| Phenylalanine | MSI 1 | Identified metabolite | 0.005075 | 0.00391–0.018777 |
| Phosphate | MSI 1 | Identified metabolite | 0.364431 | 0.2632–0.551825 |
| Propanedioic acid | MSI 1 | Identified metabolite | 0.027988 | 0.022338–0.079588 |
| Propanoic acid | MSI 1 | Identified metabolite | 0.007864 | 0.005769–0.031283 |
| Pyrogallol | MSI 1 | Identified metabolite | 0.01521 | 0.011976–0.058792 |
| Pyroglutamic acid | MSI 1 | Identified metabolite | 0.008193 | 0.007551–0.023548 |
| Quininic acid | MSI 1 | Identified metabolite | 0.012404 | 0.010673–0.041556 |
| Rhamnose I | MSI 1 | Identified metabolite | 0.01137 | 0.009043–0.042859 |
| Rhamnose II | MSI 1 | Identified metabolite | 0.044031 | 0.034427–0.156369 |
| Ribitol | MSI 1 | Identified metabolite | 0.00735 | 0.005784–0.027877 |
| Ribose I | MSI 1 | Identified metabolite | 0.007646 | 0.006704–0.032172 |
| Ribose II | MSI 1 | Identified metabolite | 0.005655 | 0.004535–0.021685 |
| Ribose III | MSI 1 | Identified metabolite | 0.010671 | 0.008577–0.040508 |
| Sarcosine | MSI 1 | Identified metabolite | 0.019407 | 0.017042–0.073577 |
| Scyllo-Inositol | MSI 1 | Identified metabolite | 0.018219 | 0.015776–0.072309 |
| Sebacic acid | MSI 1 | Identified metabolite | 0.018044 | 0.0164–0.063526 |
| Serine | MSI 1 | Identified metabolite | 0.033435 | 0.028188–0.07276 |
| Stearic acid | MSI 1 | Identified metabolite | 0.066846 | 0.053321–0.125773 |
| Succinic acid | MSI 1 | Identified metabolite | 0.013401 | 0.011128–0.054101 |
| Sucrose | MSI 1 | Identified metabolite | 0.01135 | 0.008696–0.040185 |
| Sugar_1 | MSI 3 | Class-level annotation | 0.011125 | 0.008472–0.040955 |
| Sugar_10 | MSI 3 | Class-level annotation | 0.015093 | 0.01205–0.06005 |
| Sugar_11 | MSI 3 | Class-level annotation | 0.011824 | 0.010332–0.04538 |
| Sugar_12 | MSI 3 | Class-level annotation | 0.008688 | 0.007058–0.032583 |
| Sugar_13 | MSI 3 | Class-level annotation | 0.008453 | 0.006836–0.033603 |
| Sugar_14 | MSI 3 | Class-level annotation | 0.01048 | 0.008288–0.041634 |
| Sugar_15 | MSI 3 | Class-level annotation | 0.00435 | 0.003514–0.017201 |
| Sugar_16 | MSI 3 | Class-level annotation | 0.006233 | 0.005535–0.020926 |
| Sugar_17 | MSI 3 | Class-level annotation | 0.005728 | 0.004519–0.02135 |
| Sugar_18 | MSI 3 | Class-level annotation | 0.006471 | 0.005051–0.025342 |
| Sugar_2 | MSI 3 | Class-level annotation | 0.003616 | 0.003025–0.014446 |
| Sugar_20 | MSI 3 | Class-level annotation | 0.005763 | 0.005036–0.024383 |
| Sugar_21 | MSI 3 | Class-level annotation | 0.007401 | 0.005991–0.026865 |
| Sugar_21.1 | MSI 3 | Class-level annotation | 0.081314 | 0.058317–0.238876 |
| Sugar_3 | MSI 3 | Class-level annotation | 0.00862 | 0.007297–0.032698 |
| Sugar_4 | MSI 3 | Class-level annotation | 0.013778 | 0.011136–0.052542 |
| Sugar_5 | MSI 3 | Class-level annotation | 0.00961 | 0.007933–0.03824 |
| Sugar_6 | MSI 3 | Class-level annotation | 0.016427 | 0.010717–0.0311 |
| Sugar_7 | MSI 3 | Class-level annotation | 0.009767 | 0.00738–0.036879 |
| Sugar_8 | MSI 3 | Class-level annotation | 0.006983 | 0.005621–0.027499 |
| Sugar_9 | MSI 3 | Class-level annotation | 0.004627 | 0.003541–0.017294 |
| Tagatose | MSI 1 | Identified metabolite | 0.014072 | 0.011543–0.049136 |
| Tartaric acid | MSI 1 | Identified metabolite | 0.014936 | 0.01221–0.055785 |
| Threonic acid | MSI 1 | Identified metabolite | 0.007786 | 0.00603–0.024342 |
| Threonine | MSI 1 | Identified metabolite | 0.053844 | 0.050203–0.136361 |
| Thymol | MSI 1 | Identified metabolite | 0.010272 | 0.007414–0.031392 |
| Triethylene glycol | MSI 1 | Identified metabolite | 0.011939 | 0.010274–0.049921 |
| Turanose | MSI 1 | Identified metabolite | 0.592512 | 0.458296–0.811178 |
| Tymidine | MSI 1 | Identified metabolite | 0.01042 | 0.009008–0.036151 |
| Unknown_1 | MSI 4 | Unknown feature | 0.007518 | 0.006202–0.029821 |
| Unknown_10 | MSI 4 | Unknown feature | 0.009899 | 0.008382–0.032625 |
| Unknown_11 | MSI 4 | Unknown feature | 0.016069 | 0.014009–0.05227 |
| Unknown_12 | MSI 4 | Unknown feature | 0.006953 | 0.006137–0.023693 |
| Unknown_13 | MSI 4 | Unknown feature | 0.006288 | 0.005737–0.022229 |
| Unknown_14 | MSI 4 | Unknown feature | 0.011092 | 0.009951–0.039888 |
| Unknown_15 | MSI 4 | Unknown feature | 0.010747 | 0.009746–0.038216 |
| Unknown_16 | MSI 4 | Unknown feature | 0.015341 | 0.012155–0.04094 |
| Unknown_17 | MSI 4 | Unknown feature | 0.015232 | 0.012263–0.035574 |
| Unknown_18 | MSI 4 | Unknown feature | 0.006714 | 0.005964–0.020542 |
| Unknown_19 | MSI 4 | Unknown feature | 0.010753 | 0.009892–0.03571 |
| Unknown_2 | MSI 4 | Unknown feature | 0.011591 | 0.009421–0.045811 |
| Unknown_20 | MSI 4 | Unknown feature | 0.005568 | 0.005199–0.018733 |
| Unknown_21 | MSI 4 | Unknown feature | 0.017652 | 0.014353–0.099363 |
| Unknown_22 | MSI 4 | Unknown feature | 0.021501 | 0.017633–0.072354 |
| Unknown_23 | MSI 4 | Unknown feature | 0.048209 | 0.020432–0.075937 |
| Unknown_3 | MSI 4 | Unknown feature | 0.009845 | 0.008081–0.034915 |
| Unknown_4 | MSI 4 | Unknown feature | 0.042532 | 0.035335–0.139425 |
| Unknown_5 | MSI 4 | Unknown feature | 0.016237 | 0.013064–0.041112 |
| Unknown_6 | MSI 4 | Unknown feature | 0.008489 | 0.007506–0.028799 |
| Unknown_7 | MSI 4 | Unknown feature | 0.011256 | 0.010434–0.035214 |
| Unknown_8 | MSI 4 | Unknown feature | 0.005307 | 0.004794–0.018963 |
| Unknown_9 | MSI 4 | Unknown feature | 0.00526 | 0.00467–0.01905 |
| Urea | MSI 1 | Identified metabolite | 0.179934 | 0.074749–0.352759 |
| Valeric acid | MSI 1 | Identified metabolite | 0.015653 | 0.012941–0.05717 |
| Valine | MSI 1 | Identified metabolite | 0.030044 | 0.026779–0.083008 |
| Xylonic acid | MSI 1 | Identified metabolite | 0.004849 | 0.00383–0.018252 |
| Xylose | MSI 1 | Identified metabolite | 0.018271 | 0.014723–0.059023 |

**S9. Supplementary Statistical Analysis: Multivariable Regression of Cardiac Remodeling**

To further investigate the relationship between weight loss and cardiac remodeling, we performed a multivariable linear regression analysis using ΔLVMI as the dependent variable.

Independent variables included ΔBMI, baseline LVMI, age, and sex. This model was designed to assess whether the degree of weight loss independently explained variability in cardiac remodeling.

The analysis was conducted on complete cases (N = 37). Regression coefficients were estimated using ordinary least squares (OLS), and heteroscedasticity-robust standard errors (HC3) were additionally computed to ensure robustness of the results.

In this model, ΔBMI was not significantly associated with ΔLVMI (β = -1.31, p = 0.29), whereas baseline LVMI emerged as a strong and statistically significant predictor (β = -0.55, p < 0.001). Age and sex were not significantly associated with remodeling outcomes.

These findings indicate that variability in cardiac remodeling cannot be explained solely by the magnitude of weight loss, and support the presence of additional biological determinants, including baseline cardiac structure and metabolic factors.

Given the sample size and observational design, this analysis should be interpreted as supportive rather than confirmatory.

**Supplementary Table S9. Multivariable linear regression analysis of factors associated with changes in left ventricular mass index (ΔLVMI).** The model included ΔBMI, baseline LVMI, age, and sex as independent variables. Regression coefficients are reported with heteroscedasticity-robust standard errors (HC3), 95% confidence intervals, and p-values. ΔBMI was not significantly associated with ΔLVMI, whereas baseline LVMI was the primary determinant of remodeling response.

| Variable | β coefficient | Standard Error (HC3) | 95% CI | p-value |
| --- | --- | --- | --- | --- |
| Intercept | 28.67 | 12.32 | 3.59 to 53.76 | 0.026 |
| ΔBMI | -1.31 | 1.22 | -3.79 to 1.17 | 0.290 |
| Baseline LVMI | -0.55 | 0.12 | -0.80 to -0.31 | <0.001 |
| Age | 0.04 | 0.18 | -0.34 to 0.41 | 0.848 |
| Sex | 0.82 | 6.20 | -11.80 to 13.44 | 0.895 |

**Supplementary Figure S4. Association between weight loss and cardiac remodeling before and after adjustment for clinical covariates.** (A) Scatter plot illustrating the unadjusted relationship between change in body mass index (ΔBMI) and change in left ventricular mass index (ΔLVMI) from baseline to 4 weeks. The solid line represents the linear regression fit with 95% confidence interval shading. (B) Partial regression plot showing the association between ΔBMI and ΔLVMI after adjustment for baseline LVMI, age, and sex. Residuals were obtained from linear models excluding ΔBMI (for ΔLVMI) and excluding ΔLVMI (for ΔBMI), allowing visualization of the independent contribution of weight loss. The lack of a significant adjusted association supports that early cardiac remodeling variability is not explained by the magnitude of weight loss alone.

**Figure S5. Longitudinal trajectories of relative wall thickness (RWT) and transitions across remodeling categories.** (A) Individual trajectories of relative wall thickness (RWT) across timepoints (T0, T4, T12, T24). Each thin line represents a single patient, while the bold line indicates the mean trajectory. The dashed horizontal line represents the clinical threshold for concentric remodeling (RWT = 0.42). (B) Alluvial representation of patient transitions across the RWT threshold over time. The width of each flow is proportional to the number of individuals transitioning between categories (“Above threshold” vs “Below threshold”) between consecutive timepoints. This visualization highlights the dynamic and heterogeneous nature of early geometric remodeling following bariatric surgery.

**Figure S6. PLS-DA trajectories of systemic metabolomic profiles across time.** PLS-DA score plot showing longitudinal trajectories of metabolomic profiles following bariatric surgery. Each point represents an individual sample projected onto the first two latent variables of the PLS-DA model, after median normalization, log transformation, and autoscaling. Thin grey lines indicate individual patient trajectories across timepoints (T0, T4, T12, T24). Colored lines represent the mean trajectory for each group (No remodeling vs Yes remodeling). Ellipses denote group-wise dispersion at each timepoint (95% confidence intervals). These analyses are exploratory and intended to visualize temporal evolution of metabolomic profiles rather than provide inferential classification.

**Figure S7. LASSO regression–derived metabolic features associated with cardiac remodeling indices at baseline (T0).** Horizontal bar plot showing the top metabolites selected by LASSO regression as predictors of cardiac geometry–related parameters, including left ventricular mass index (LVMI) and relative wall thickness (RWT). Positive coefficients (right side) indicate metabolites positively associated with the modeled outcome, whereas negative coefficients (left side) indicate inverse associations. The selected features include metabolites involved in energy metabolism, amino acid turnover, and lipid handling, as well as several unannotated compounds. The predominance of carbohydrate-related and organic acid metabolites suggests a potential role of systemic metabolic regulation in shaping myocardial structural properties. Given the exploratory nature of the analysis, these results should be interpreted as associative and hypothesis-generating.

**Supplementary**

**Figure S8. Radial variability plot of longitudinal metabolite fluctuations across time points.**

The circular plot summarizes the longitudinal variability of serum metabolites from baseline (T0) to 24 weeks (T24), grouped into five major biochemical classes: amino acids (green), fatty acids (blue), short-chain fatty acids (SCFAs) (violet), sugars (red), and other metabolites (yellow). Each bar represents an individual metabolite, arranged along the circular axis according to its class membership.

Colored bars indicate the percentage variability across all time points (T0–T24), while grey bars represent the variability observed specifically at 4 weeks (T4). This comparative visualization highlights molecular groups whose short-term variability (T4) remains below the overall longitudinal fluctuation observed throughout follow-up.

Metabolites exhibiting the greatest temporal fluctuations relative to population-level variability included leucine, galacturonic acid, mannitol, thymidine, N-formylglycine, valine, isoleucine, lactic acid, 3-phenyllactic acid, and threonine, underscoring their potential role as dynamic markers of metabolic remodeling in response to the intervention.

**Figure S9. LASSO regression model identifying baseline metabolites associated with relative wall thickness (RWT).**

The horizontal bar plot displays the standardized regression coefficients obtained from the LASSO model, using baseline (T0) metabolomic data and relative wall thickness (RWT) as a continuous outcome. Metabolites with negative coefficients (purple bars) show an inverse association with RWT, whereas positive coefficients (green bars) indicate a direct relationship. Among the most influential predictors were nonanoic acid, meso-erythritol, 2-furanacetic acid, and mannose I, together with several unannotated sugar derivatives. These metabolites collectively suggest that alterations in carbohydrate and organic acid metabolism may contribute to cardiac geometric remodeling, potentially reflecting disturbances in intracellular osmotic regulation and myocardial stiffness.
